# An Automated Methodology for Non-targeted Compositional Analysis of Small Molecules in High Complexity Environmental Matrices Using Coupled Ultra Performance Liquid Chromatography Orbitrap Mass Spectrometry

**DOI:** 10.1021/acs.est.0c08208

**Published:** 2021-05-18

**Authors:** Kelly L. Pereira, Martyn W. Ward, John L. Wilkinson, Jonathan Brett Sallach, Daniel J. Bryant, William J. Dixon, Jacqueline F. Hamilton, Alastair C. Lewis

**Affiliations:** † Wolfson Atmospheric Chemistry Laboratories, Department of Chemistry, University of York, York YO10 5DD, U.K.; ‡ Department of Environment and Geography, University of York, York YO10 5NG, U.K.

**Keywords:** ultrahigh-resolution mass spectrometry, non-targeted analysis, Compound Discoverer, liquid chromatography−mass spectrometry

## Abstract

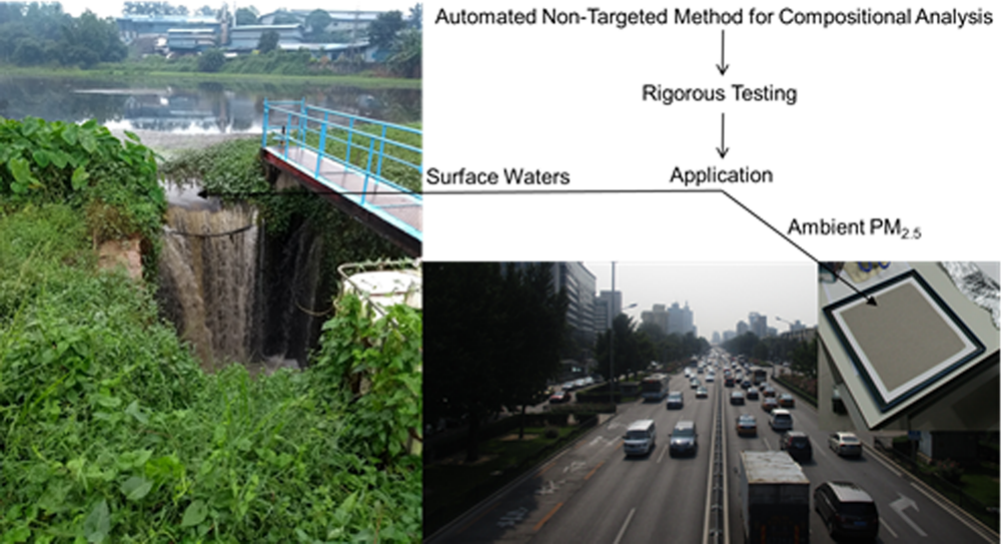

The life-critical matrices of air and water are among the most complex chemical mixtures that are ever encountered. Ultrahigh-resolution mass spectrometers, such as the Orbitrap, provide unprecedented analytical capabilities to probe the molecular composition of such matrices, but the extraction of non-targeted chemical information is impractical to perform via manual data processing. Automated non-targeted tools rapidly extract the chemical information of all detected compounds within a sample dataset. However, these methods have not been exploited in the environmental sciences. Here, we provide an automated and (for the first time) rigorously tested methodology for the non-targeted compositional analysis of environmental matrices using coupled liquid chromatography–mass spectrometric data. First, the robustness and reproducibility was tested using authentic standards, evaluating performance as a function of concentration, ionization potential, and sample complexity. The method was then used for the compositional analysis of particulate matter and surface waters collected from worldwide locations. The method detected >9600 compounds in the individual environmental samples, arising from critical pollutant sources, including carcinogenic industrial chemicals, pesticides, and pharmaceuticals among others. This methodology offers considerable advances in the environmental sciences, providing a more complete assessment of sample compositions while significantly increasing throughput.

## Abstract 2

An automated non-targeted method for environmental matrices has been developed and rigorously tested. The method provides a more complete assessment of sample compositions, while significantly increasing throughput.

## Introduction

Environmental pollution accounts for ∼9 million premature deaths per annum.^1^ Of these deaths, 4.2 million are attributed to ambient particulate matter (PM) and a further 1.8 million to unsafe water sources and sanitation.^2^ Air and water matrices typically contain 10^3^–10^5^ pollutants, with a diverse range of chemical functionalities and concentrations.^3,4^ The sheer number of pollutants present in the environment, their varied sources, chemical functionalities, and concentrations make the compositional analysis of these species a formidable analytical challenge. Regulatory and enforcement bodies assess air and water pollution via the measurement of prescribed lists of targeted compounds using predefined analytical methods.^5,6^ While the number of regulated pollutants has increased in recent years, these targeted compounds represent a tiny proportion of the thousands of pollutants actually present. Consequently, most pollutants in air and water go undetected, including potentially hazardous site-specific and emerging contaminants.^7^

Fourier transform mass spectrometers (FTMS) offer unprecedented capabilities to probe the molecular composition of highly complex matrices, with resolving powers greater than ∼10^5^ full width at half-maximum at a mass-to-charge ratio (*m/z*) of 200, with mass accuracies <2 parts per million. There are only two FTMS: the Orbitrap (ThermoFisher Scientific) and the Fourier transform ion-cyclotron resonance (Bruker Daltonics). The molecular identification of unknowns in complex matrices requires a mass analyzer with high mass accuracy to reduce the ambiguity in molecular formula assignments and high mass resolution to minimize the overlap of isobaric species. Chromatographic separation provides additional chemical specificity, allowing structural isomers to be distinguished. These techniques however generate significant volumes of highly complex data, resulting in users targeting limited lists of compounds to reduce data complexity and increase throughput. These targeted approaches, analogous to regulatory methods, provide severely limited compositional information.

Automated non-targeted screening tools can overcome these challenges, rapidly extracting the chemical information of all detected compounds within a sample dataset, highlighting background artifacts, providing molecular formula assignments (among other information) and probable structure through mass spectral library screening. This compositional information is incredibly beneficial and essential in several scientific disciplines.^8−12^ While automated non-targeted screening can reduce data analysis time from months to hours, these methods have not been exploited in the environmental sciences. Non-targeted tools have been designed mainly for the analysis of metabolites and proteins (^13−16^ and references therein). These tools lack the chemical metrics frequently used in the environmental sciences to aid in the identification of pollutant sources (e.g*.*, elemental ratios,^17^ average carbon oxidation state,^18^ aromaticity index,^19^ and various compositional groupings^20−22^) and may in part account for the slow uptake within the environmental domain. Further, to our knowledge, no studies have investigated the performance and reproducibility of these methods for the compositional analysis of trace-level compounds in environmental matrices.

Here, we present an automated methodology for the non-targeted compositional analysis of environmental matrices using coupled ultrahigh-performance liquid chromatography–Orbitrap MS. The method consists of a bespoke workflow developed in the instrument manufacturers’ commercial software, providing seamless integration with the instrumentation and automated screening of the largest tandem mass spectral database (mzCloud, www.mzcloud.org; not possible with other platforms) and a custom-built data processing program. The data program further advances the workflow capabilities, including a more rigorous approach for the removal of artifacts from the sample data and the automated calculation of numerous environmental chemical metrics and groupings to aid in compositional interpretation and allow for the rapid comparison of sample compositions. The method is applicable to any ThermoFisher Scientific FTMS (i.e., FTMS market leader and includes all Orbitrap designs). First, we test the ability of the method to detect, identify, and integrate authentic standards frequently observed in environmental matrices, evaluating performance as a function of analyte concentration, ionization potential, and sample complexity. We then evaluate the performance for the analyses of PM and surface waters collected from worldwide locations.

## Materials and Methods

### Standards

Sixty authentic standards were used to test the detection, identification, and integration capabilities of the non-targeted data processing method. The compound names, manufacturer, and purity of the standards can be found in Table S1. The standards were prepared at 1 ppm and in mixtures at concentrations ranging from 5 ppm to 0.5 ppb. Two compounds, furan-2,5-dione and 3-methylfuran-2,5-dione, were excluded from the standard mixture to prevent the formation of their acid counterparts, which were also included. Standards were prepared in 50:50 methanol/water (optima, LC–MS grade, ThermoFisher Scientific) for analysis. Calibrations were performed for any of the standards identified in the environmental samples and consisted of a minimum of five concentrations, with three replicate measurements per concentration.

### Ambient Particulate Matter

Samples were collected at the Institute of Atmospheric Physics (IAP), Chinese Academy of Sciences in Beijing, China (Lat. 39°58′28″ N, Long. 116°22’15″ E) onto quartz fiber filters at a flow rate of 1.33 m^3^/min using a HiVol sampler with a PM_2.5_ inlet (model 3000, Ecotech). The sampler was positioned on the roof of the IAP building ∼8 m above ground level. Filters were pre-conditioned in a furnace at 500 °C for 5 h to remove any volatiles before use. The sampling dates and times are shown in Table S2. After sample collection, each filter was wrapped in foil to minimize potential photolysis degradation and stored in a freezer at −20 °C. Filters were shipped in dry ice to the University of York for analysis. Samples were prepared using the methodology detailed in Bryant et al. (2019).^23^ Briefly, 1/8th of each filter was extracted into 4 mL of water, left for 2 h at room temperature, and sonicated for 30 min. The aqueous extract was then filtered through a 0.22 μm filter membrane, evaporated to dryness (model V10, Biotage, Sweden), and reconstituted in 1 mL of 50:50 methanol/water. A procedural blank was also prepared, consisting of a blank pre-conditioned filter subjected to the same sample extraction and preparation procedure.

### Surface Waters

Samples were collected from China, India, and Sri Lanka. Full details of the sample collection locations are shown in Table S3. Samples were collected using the protocol described in Wilkinson et al. (2019).^24^ Briefly, 10 mL aliquots of water were collected via grab sampling, filtered through a 0.45 μm glass microfiber filter membrane, and shipped in dry ice to the University of York where they were stored at −80 °C. Prior to analysis, 800 μL of each sample was evaporated to dryness and reconstituted in 80 μL of 50:50 methanol/water. A procedural blank was also prepared consisting of 800 μL of high-purity water, subjected to the same sample preparation procedure.

### Instrument and Data Analysis

Full method details can be found in the Supporting Information; a brief summary is given here. The standards and samples were analyzed using ultrahigh-performance liquid chromatography coupled to an ultrahigh-resolution mass spectrometer (Dionex 3000-QExactive Orbitrap, UPLC-MS, ThermoFisher Scientific). Data were analyzed using the non-targeted method, consisting of a bespoke workflow developed in the framework Compound Discoverer (version 2.1, ThermoFisher Scientific) and a custom-built data processing program developed in Python (version 3.7). The workflow is shown in Figure S1. The workflow was designed to, (i) align the chromatographic retention times of input data files, (ii) extract all chromatographic peaks which met set criteria (see below), (iii) assign the molecular formula for each compound and, (iv) screen the MS^2^ data against an in-house built and commercial library (mzCloud) for possible compound identification. The in-house MS^2^ library was developed in the software package mzVault (version 2.0, ThermoFisher Scientific, supplied with Compound Discoverer) using the spectra obtained from the analysis of the 60 individually prepared standards. Chromatographic peaks were detected if the molecular species had a signal-to-noise ratio >3, a minimum peak intensity of 3 × 10^4^ and was detected in a minimum of three consecutive scans. Molecular formula assignments were allowed unlimited C, H, O atoms and up to 5 N atoms, 2 S atoms and 3 Cl atoms (surface water analysis only). In positive ionization mode, 2 Na atoms and 1 K atom were also allowed. Molecular formulae were only assigned if the isotopic intensity tolerance was within ±30% of the theoretical isotopic abundance and the mass tolerance was <3 ppm. The software also screens the sample data for the detection of common electrospray ionization (ESI) artifacts. The list of ESI artifacts is shown in Table S4. Where multiple adducts are detected, the software will group these species and report the data for only one adduct, typically [M + H]^+^ or [M – H]^−^ (i.e., user-specified preferred adduct). All other artifacts are removed from the sample data. Specific workflows were developed for the analysis of positive and negative ionization mode data. The data program was developed to perform additional screening functions and calculations, which could not be performed in Compound Discoverer. The program was designed to (i) tabulate the workflow output into a user-friendly format, (ii) remove system (i.e., solvent blanks) and sample preparation (i.e., method procedural blanks) artifacts using a more rigorous approach (designed for complex matrices, see the Supporting Information, “Removal of Artifacts”), (iii) remove components with unassigned or erroneous molecular formulae, (iv) perform chemical metric calculations to aid in the identification of pollutant sources, and (v) output the data using various chemical groupings to allow for the rapid comparison of sample compositions. All manual data processing was performed in the software Freestyle (version 1.1, ThermoFisher Scientific). The workflows, in-house MS^2^ library, and data program can be downloaded from a public depository (doi:10.5281/zenodo.4701800).

## Results

### Initial Software Testing

Sixty authentic standards were initially used to test the detection, identification, and integration capabilities of the non-targeted method. The standards contained a diverse range of chemical functionalities (e.g., carboxylic acids, carbonyls, alcohols, aromatics, nitrophenols) representing the types of compounds often observed in environmental samples. The molecular weight (MW) of the standards ranged from 96 to 232, with an average oxygen-to-carbon (O/C) ratio of 0.47 and a carbon number range of C_3_ to C_15_. First, the ability of the method to detect and identify sample components was tested using the individually prepared standards at a concentration of 1 ppm. The sample complexity was then increased, testing the method’s ability to detect, identify, and integrate the standards in a mixture prepared at concentrations between 5 ppm to 0.5 ppb. The standards were grouped into the types of molecular species detected (e.g., deprotonated, protonated, sodiated), investigating whether the performance was affected by negative ionization mode or adduct formation in positive ionization mode.

The 60 standards were initially characterized via manual data processing, recording if the standard was detected, the ionization mode and type of molecular species, the chromatographic retention time, and the MS^2^ fragmentation spectrum, which was recorded in the in-house library. This data was then used to evaluate the performance of the non-targeted method. In total, 45 standards were detected in negative ionization mode as deprotonated molecular species [M – H]^−^*.* In positive ionization mode, 28 standards were detected as protonated molecular species [M + H]^+^ and a further 26 standards were detected as sodiated molecular species [M + Na]^+^. Potassiated molecular species [M + K]^+^ were observed for some standards but represented less than ∼1% of the total precursor signal intensity and were subsequently excluded from further analysis. The detected molecular species and their retention times (determined via manual analysis) are shown in Table S5.

The performance of the non-targeted method to detect the chromatographic peaks and assign the molecular formulae and compound names (i.e., molecular identities, determined from MS^2^ library screening) of the standards is shown in Figure S2. The non-targeted method successfully detected the chromatographic peaks and correctly identified the molecular formulae for all 45 [M – H]^−^ and 28 [M + H]^+^ standards (Figure S2A,B). The ability of the method to detect and identify [M + Na]^+^ standards is shown in Figure S2C. The method detected the chromatographic peaks and provided the molecular formulae for 15 out of 26 [M + Na]^+^ standards (i.e., 58%). The software appears to initially search for [M + H]^+^ species in positive ionization mode. If detected, the software then searches for [M + Na]^+^ (see the Supporting Information, “Sodium Adduct Detection” for further information). Consequently, any compounds that are exclusively observed as [M + Na]^+^ in positive ionization mode are not detected by the software. This parameterization cannot be changed by the user (i.e., a part of the underlying software algorithms), demonstrating the importance of testing automated non-targeted methods to provide a fundamental understanding of their potential limitations prior to use.

The number of standards identified by the non-targeted method using the in-house and commercial library is shown in Figure S2. The MS^2^ spectrum of some standards was not obtained during analysis, preventing molecular identification using library screening (shown as “omitted” data in Figure S2). Excluding those standards where no MS^2^ spectra were obtained, the method provided the molecular identity for 84% of the [M – H]^−^ (34 out of a possible 44) and 95% of the [M + H]^+^ (21 out of 22) standards. The software was unable to provide the molecular identities of the standards exclusively observed as [M + Na]^+^ in positive ionization mode as the chromatographic peaks were not detected. Excluding these standards, the non-targeted method provided the molecular identity for 73% of the [M + Na]^+^ standards (11 out of a possible 15). Overall, the in-house library provided the identities for 85% (69 out of 89) of the total number of molecular species (i.e., deprotonated, protonated, and sodiated standards). The commercial library contained spectra for 24 of the 60 standards in its database and subsequently provided the identity of fewer standards in comparison to the in-house library, identifying only 21% (18 out of 89) of the total number of molecular species.

### Increasing Sample Complexity

**Figure 1:**
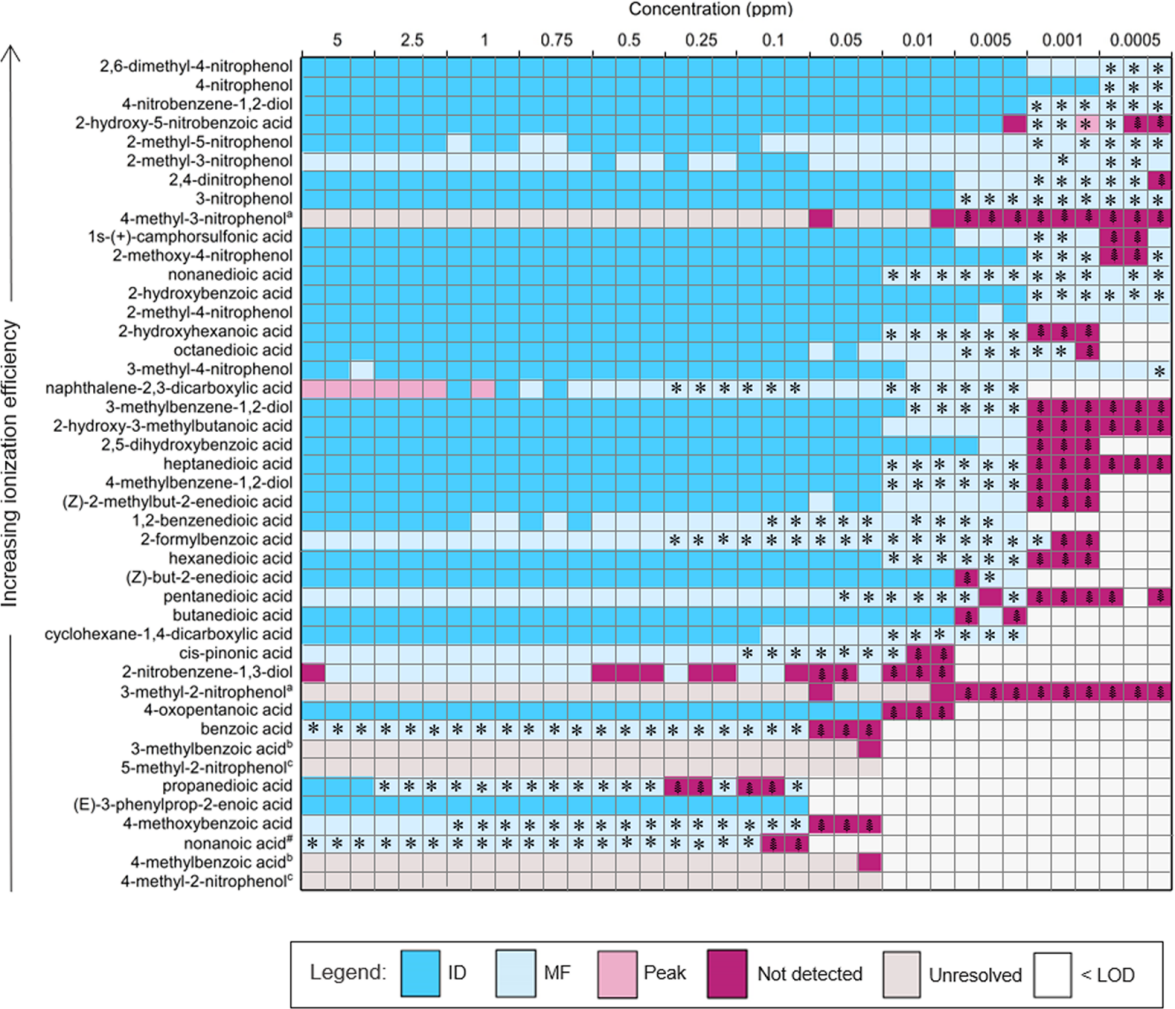
Performance of the non-targeted method to detect and identify [M – H]^−^ species in the standard mix prepared at various concentrations. The plot displays whether the compound names (ID), molecular formulae (MF), and chromatographic peak (Peak) were identified. Each box represents one measurement, with three replicate measurements performed for each concentration. The asterisks indicate that no MS^2^ data was recorded during analysis preventing molecular identification. The palm branches indicate that the chromatographic peak cannot be detected due to the use of unit mass resolution. Isomeric species that could not be resolved via manual or automated data processing are shown in gray. Letters correspond to the groups of isomeric species which could not be resolved; ^a^ = 3-methyl-2-nitrophenol and 4-methyl-3-nitrophenol. ^b^ = 3-methylbenzoic acid and 4-methylbenzoic acid, ^c^ = 5-methyl-2-nitrophenol and 4-methyl-2-nitrophenol. The hash symbol indicates that the in-house library contains no MS^2^ spectra for this standard.

The 60 standards (excluding two compounds, see Materials and Methods) were combined into a single mixture, prepared at various concentrations, and used as a proxy to test the performance of the method. The chromatographic peak areas of the individually prepared standards (at the same concentration) were integrated and used as a metric to describe the ionization efficiency of each molecular species, allowing the standards to be ordered by increasing ionization efficiency. The ability of the method to detect and identify each compound in the standard mixtures, in negative and positive ionization mode, is shown in Figure 1 and Figure S3, respectively. Three replicate sample injections were performed for each standard mixture to investigate the reproducibility of the non-targeted method to report the same result. Several isomeric compounds could not be resolved in the standard mixtures via manual or automated data processing due to co-elution and are shown in Figure 1 and Figure S3 as “unresolved”.

From Figure 1 and Figure S3A, it can be observed that the non-targeted method struggled to detect some standards at the lowest observable concentration and was particularly evident with decreasing ionization efficiency. There are four main parameters in the non-targeted method that control chromatographic peak detection, particularly at low concentrations. The majority of these parameters can be found in the “detect unknown compounds” node (Figure S1). However, the workflow is based on a hierarchical structure. Therefore, any parameters prior to and including the detect unknown compounds node can affect chromatographic peak detection. The four main parameters include the signal-to-noise (S/N) threshold (select spectra and detect unknown compounds node), minimum peak intensity, isotopic intensity tolerance, and the minimum scans per peak. Each parameter was individually tested, increasing or decreasing the value to remove any restrictions. Excluding the S/N threshold in the select spectra node, which incorrectly determined some standards to be <LOD (see the Supporting Information, “Software Notes”), all other parameters did not improve the chromatographic peak detection capabilities.

The non-detection of the low concentration species can instead be explained by considering the mass resolution used for chromatographic peak detection. The software uses unit mass resolution (i.e., an integer value) for chromatographic peak detection. This is incredibly beneficial for reducing software processing time. However, this does not utilize the accurate mass capability of the instrumentation, capable of achieving >6 decimal places. The use of unit mass resolution (particularly for the analysis of complex matrices) will not be able to fully resolve chromatographic peaks from other components in the sample, resulting in lower S/N ratios, increasing the number of chromatographic peaks, which are determined to be <LOD. For example, deprotonated 3-methylbenzene-1,2-diol in the 0.001 ppm standard mixture had an S/N ratio of 1.89 using a unit mass range of *m/z* 123 to 124 (determined via manual analysis). Using an accurate mass range of *m/z* 123.0450 to 123.0460, deprotonated 3-methylbenzene-1,2-diol had an S/N ratio of 20.8 (a factor of 11 increase), accounting for the difficulties in the detection of low concentration species. Analogous to sodium adduct detection, the mass resolution used for chromatographic peak detection cannot be controlled by the user (a part of the underlying software algorithms).

The number of chromatographic peaks, which could not be detected using unit mass resolution, is shown in Figure 1 and Figure S3 (demonstrated using manual analysis). Unit mass resolution accounted for the non-detection of 86 and 76% of the [M – H]^−^ and [M + H]^+^ standards, respectively. It is worth noting that protonated 4-methoxybenzoic acid co-eluted with an isomeric fragment of (4-formyl-2-methoxyphenol)acetate, accounting for the difficulty in the detection of this species. Excluding protonated 4-methoxybenzoic acid, unit mass resolution accounted for the non-detection of all [M + H]^+^ standards. Conversely, only 26% of the non-detected [M + Na]^+^ standards could be attributed to the use of unit mass resolution for chromatographic peak detection. The non-detected [M + Na]^+^ standards (as previously discussed) is primarily due to the inability of the software to assign sodium adduct formation if the protonated molecular species is not detected, accounting for the non-detection of levoglucosan, 2-hydroxyhexanoic acid, 2-hydroxy-3-methylbutanoic acid, and 2,3-diacetyloxypropyl acetate, which were exclusively observed as [M + Na]^+^ in positive ionization mode, as shown in Figure S3B.

A summary of the method’s performance to detect and identify each compound in the standard mixtures is shown in Figure S4. Overall, the non-targeted method detected the chromatographic peaks and identified the molecular formulae for 91.6 ± 1.0% (mean ± variation from the mean) of the total number of [M – H]^−^ and [M + H]^+^ standards and provided the identity for 70 and 59%, respectively. For [M + Na]^+^ standards, the method detected the chromatographic peaks and provided the molecular formulae for 57% and correctly identified 28%. The method consistently reported the same result for each standard in the replicate sample injection measurements and data analyses, with 92% of all molecular species displaying no variation (Figure 1 and Figure S3). The largest variation in the detection and identification of the standards was observed at the lowest concentrations, likely the result of low-intensity MS^2^ spectra and/or compounds close to the LOD (i.e., instrument variation close to the software “cut-off” values). The integration capabilities of the non-targeted method are shown in Figure S5. The chromatographic peaks of the standards were integrated via the non-targeted method and manual data processing, allowing calibration graphs to be plotted and the integration capabilities of the two methods to be compared. Both methods displayed good agreement with an *R*^2^ of 0.9993 and a slope of 1.14 ± 0.007. The primary limitation observed with increasing sample complexity was poor chromatographic separation of isomeric species. Where two unresolved isomeric compounds were present, the non-targeted method typically reported the detection of only one of the standards, usually the most abundant. This limitation however cannot be attributed to the non-targeted method; those compounds could not be resolved using either manual or automated data processing and is ultimately dependent upon the analytical method (i.e., a balance between throughput and chromatographic resolution).

### Analysis of Ambient Particulate Matter

Here, we use the non-targeted method to investigate the chemical composition of eight ambient PM samples collected in Beijing, China, evaluating the method’s performance through the comparison of detected pollutants, their sources, and abundance (including quantitative measurements) with literature observations and modeled data. The method incorporates two screening approaches: (i) non-targeted, where the chemical information of all detected compounds in each sample is reported, and (ii) targeted, which uses the in-house library to screen the samples for the identification of the 60 authentic standards initially used to test the method. Manual data analysis was also used to test whether the non-targeted method had correctly reported the identification of the targeted standards in the PM samples. The molecular identification of these compounds was confirmed using the retention times and fragmentation spectra of the authentic standards. Calibrations were also performed, providing quantitative measurements. The PM samples consisted of four samples collected during the summer season and a further four collected during the winter season. Each respective season included two samples collected during the daytime and two overnight. Full details can be found in Table S2.

The non-targeted method detected between 4402 to 9655 compounds in the individual PM samples (sum of negative and positive ionization mode, see Table S6). The in-house library identified a total of 185 compounds, which included multiple identifications of the standards in each PM sample. Of the targeted compounds, 147 were quantified. The concentrations of the other 38 compounds could not be determined as the chromatographic peak areas were outside (i.e., either below or above) the measured linear calibration ranges preventing quantification. The ambient concentrations of the targeted compounds in the PM samples are shown in Tables S7 and S8. The sheer number of compounds, which can be detected using the non-targeted vs targeted approach, is shown in Figure S6, as an example for one PM sample. Of the 60 standards used for targeted identification, 20 were identified in this sample. In contrast, the non-targeted approach detected 5089 unique compounds (i.e., chemically and/or structurally different). Using the targeted approach, <1.1% of the organic PM mass (by weight) was quantified in all the samples, demonstrating the importance of using non-targeted methods in the environmental sciences to provide a more complete assessment of sample compositions.

**Figure 2:**
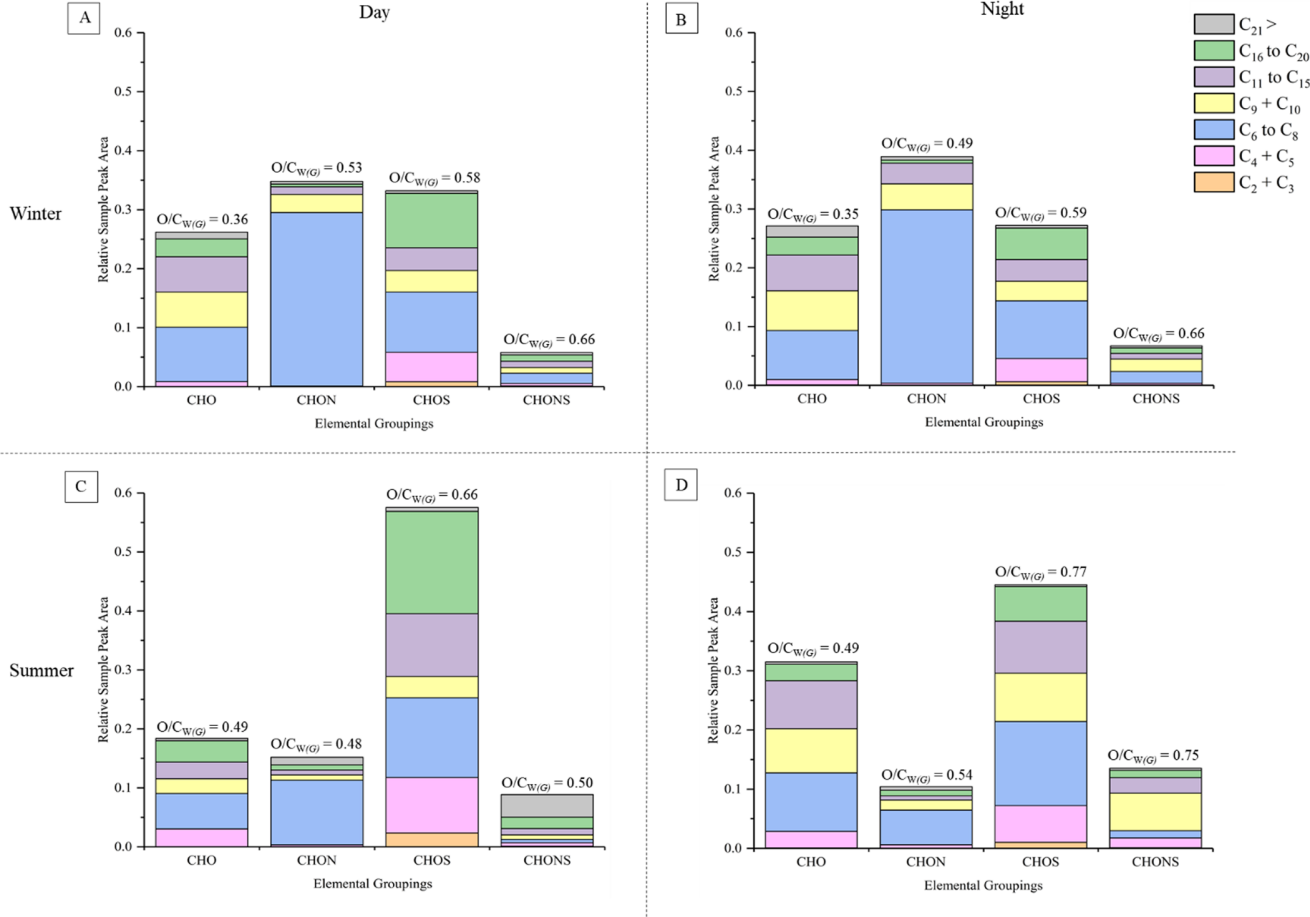
PM_2.5_ samples collected in Beijing during the winter season in the day (A) and night (B) and summer season in the day (C) and night (D), displaying all detected compounds grouped by their elemental composition and number of carbon atoms in each molecular formula, as a function of their relative sample peak area. Each plot shows the average composition of two aerosol samples collected during the same season and time of day using the data acquired from negative ionization mode. O/C_W(G)_ displays the weighted oxygen-to-carbon ratio, calculated by dividing the peak area of each elemental grouping by the total sample peak area.

To evaluate the insights, which can be obtained using non-targeted data, we compare the chemical composition of the ambient PM samples collected during summer and winter, day and night. The composition of each PM sample was separated into several groupings using the automated data processing program, see the Supporting Information, “Data Processing Program” for further information. Briefly, all detected compounds in each PM sample were grouped by the number of carbon atoms and elemental composition in their molecular formulae, relating to potential pollutant sources. The peak areas of each compound were normalized to the total peak area in each sample, allowing the relative abundance of the chemical groupings between samples to be compared, as shown in Figure 2.

Figure 2 shows that the abundance of the C_6_ to C_8_ CHON grouping is ∼3 times more significant in winter vs summer. Using the processed program data, the chemical information of the compounds, which contribute to this grouping in both PM samples, can be rapidly observed. In the winter daytime sample (sample 96, see Table S2), 220 compounds were detected in the C_6_ to C_8_ CHON grouping. 98% of these compounds had DBE/C values >0.5, suggesting that the majority are aromatic and polycyclic aromatic compounds, also supported by the commercial and in-house library matches. The compounds were relatively oxidized, with an average O/C ratio of 0.52 and MW of 177. In contrast, fewer compounds were observed in the C_6_ to C_8_ CHON grouping in the daytime summer sample (sample 261). In this sample, 77 compounds were detected, 88% which had DBE/C values >0.5. The average O/C ratio was 0.59 and MW of 184, suggesting that the winter and summer PM sample compositions are relatively similar, with the summer sample containing more oxidized and non-aromatic compounds.

Using chemical metric plots, the compositional differences between these chemical groupings can be further explored. Figure S7 shows the composition and relative abundance of each C_6_ to C_8_ CHON compound in the summer and winter samples in a DBE/C vs molecular weight space. While the winter sample contained a greater number of C_6_ to C_8_ CHON species, Figure S7 shows that the composition is dominated by ∼3 compounds, which have considerably higher abundances than in the summer sample, accounting for the differences in Figure 2. The relative abundance of several identified compounds (determined via targeted screening), including 4-nitrophenol, 3-methyl-4-nitrophenol, and 2,6-dimethyl-4-nitrophenol were comparable between winter and summer, with ratios of 1.3, 6.0, and 3.0 for winter/summer, respectively. The most abundant compound in winter was identified as 4-nitrobenzene-1,2-diol (i.e., 4-nitrocatechol). The other two abundant compounds are suggested to be methyl nitrocatechols (*t*_R_ 7.15 and 8.82), displaying the characteristic neutral losses of NO, HNO_2_, or NO_2_ and the combined loss of NO and CO.^25,26^ 4-Nitrocatechol and methyl nitrocatechols are well-known abundant oxidation products of biomass burning, formed via OH or NO_3_ oxidation of catechol or methyl catechol under medium NO*_x_* conditions.^27^ The relative abundances of 4-nitrocatechol and methyl nitrocatechol (*t*_R_ 8.82) were determined to be a factor of 62 and 266 times more significant in the winter sample, respectively. Further, methyl nitrocatechol at *t*_R_ 7.15 was not detected in the summer sample, suggesting that the differences observed in the winter C_6_ to C_8_ CHON grouping are the result of biomass burning influences, which are known to be more abundant in winter.^28−33^

Similarly, Figure 2 shows that the C_16_ to C_20_ CHOS grouping is most abundant in the daytime and the summer season. C_16_ to C_20_ CHOS groupings comprise several species that dominate the chemical composition, as shown in Figure S8. These compounds were not very oxidized (O/C ratio 0.17 to 0.19) and all displayed similar chemical properties, containing three oxygen atoms and four double bonds and differed in their molecular formula by CH_2_. The commercial library identified 4-dodecylbenzenesulfonic acid (spectral match >91% confidence), a surfactant mainly used in laundry detergent and commonly produced in a mixture of linear alkylbenzene sulfonates (LAS).^34^ An additional five abundant C_16_ to C_20_ CHOS species could also be observed, including C_17_H_28_O_3_S (MW 312, *t*_R_ 22.37, 22.49, and 22.68) and C_16_H_26_O_3_S (MW 298, *t*_R_ 21.25 and 21.53). All of these compounds displayed fragment ions *m/z* 183, 119, and 80, corresponding to characteristic LAS fragmentation patterns,^35^ supporting the tentative identification of 4-dodecylbenzenesulfonic acid by the commercial library. LASs have been observed in rainwater^36^ and fog extracts,^37^ although due to their low volatility, the observation of these compounds in PM is rare. Nevertheless, it is worth noting that the sampling site was close to a launderette, which was closed during nighttime hours, possibly accounting for the observed decrease in the nighttime LAS abundance and may indicate a new potential daytime source.

The method also tentatively identified several compounds in the PM samples used in the agricultural industry, with spectral matches >82% confidence for an insecticide (omethoate, C_5_H_12_NO_4_PS, *t*_R_ 2.11), a fungicide (triadimefon, C_14_H_16_ClN_3_O_2_, *t*_R_ 17.30), and an herbicide (acetochlor, C_14_H_20_ClNO_2_, t_R_ 18.02). Acetochlor and triadimefon are suspected carcinogenic compounds,^38−40^ and omethoate is known to be highly toxic to the aquatic environment.^40^ Interestingly, these compounds were only observed in the samples collected overnight in the summer season (samples 264 and 274, Table S2) and appear to be products of long-range transportation from air masses outside of the city (see Figure S9). These pollutants have previously been observed in PM^41−44^ and are known to correlate with agricultural activities,^44^ remaining airborne for several days following spraying,^45^ supporting these observations and modeled data.

### Analysis of Surface Waters

Finally, we use the non-targeted method for the compositional analysis of surface waters collected from seven different locations across China, India, and Sri Lanka. The sampling locations are shown in Table S3. Non-targeted screening detected between 1503 to 9165 compounds in the individual samples (sum of positive and negative ionization mode, see Table S9). In contrast to ambient PM, targeted screening using the in-house library identified fewer compounds in the surface water samples. A total of 60 standards were identified, 35 of which were quantified. The commercial library however, offered considerable advances, providing tentative molecular identifications for an additional 94 compounds with spectral matches >85% confidence. The concentrations of the targeted compounds in the surface water samples are shown in Table S10. The measured concentrations of octanedioic and nonanedioic acid, identified in all surface water samples, were found to be remarkably comparable with those recently reported in the River Rhone, France,^46^ ranging from 0.11 to 0.49 and 0.16 to 0.70 μg L^–1^, respectively.

Here, we use the chemical groupings commonly used for the compositional analysis of dissolved organic matter (DOM) but also utilize the strengths of the non-targeted method, grouping all tentatively identified compounds with spectral matches >85% confidence (assigned by the commercial library) by their potential pollutant sources, as shown in Table S11. The commercial library identified several harmful pollutants in the surface water samples, including carcinogenic industrial and agricultural chemicals (tributyl phosphate and carbendazim), active ingredients in pharmaceutical medication (e.g., anaesthetic, analgesic, antipsychotics), stimulants (caffeine; high toxicity risk for aquatic organisms^47^), potential illicit drugs (methamphetamine), and personal care products (*N*,*N*-diethyl-*m*-toluamide, DEET) among others. The identification of these pollutants in surface waters is not uncommon.^47−49^ For example, DEET is the primary ingredient used in insect repellents^50^ and an abundant and frequently detected pollutant in surface waters.^51−53^ DEET was detected in three surface water samples (S1–S3, see Table S3) and was most prominent in the industrial and wastewater effluent samples collected in Sri Lanka, representing >12% of the total sample abundance. In fact, DEET accounted for >45% of the most abundant DOM chemical grouping (i.e., highly unsaturated CHON) in the wastewater effluent sample, as shown in Figure S10, compositional information that would not have been observed without the use of non-targeted screening.

**Figure 3:**
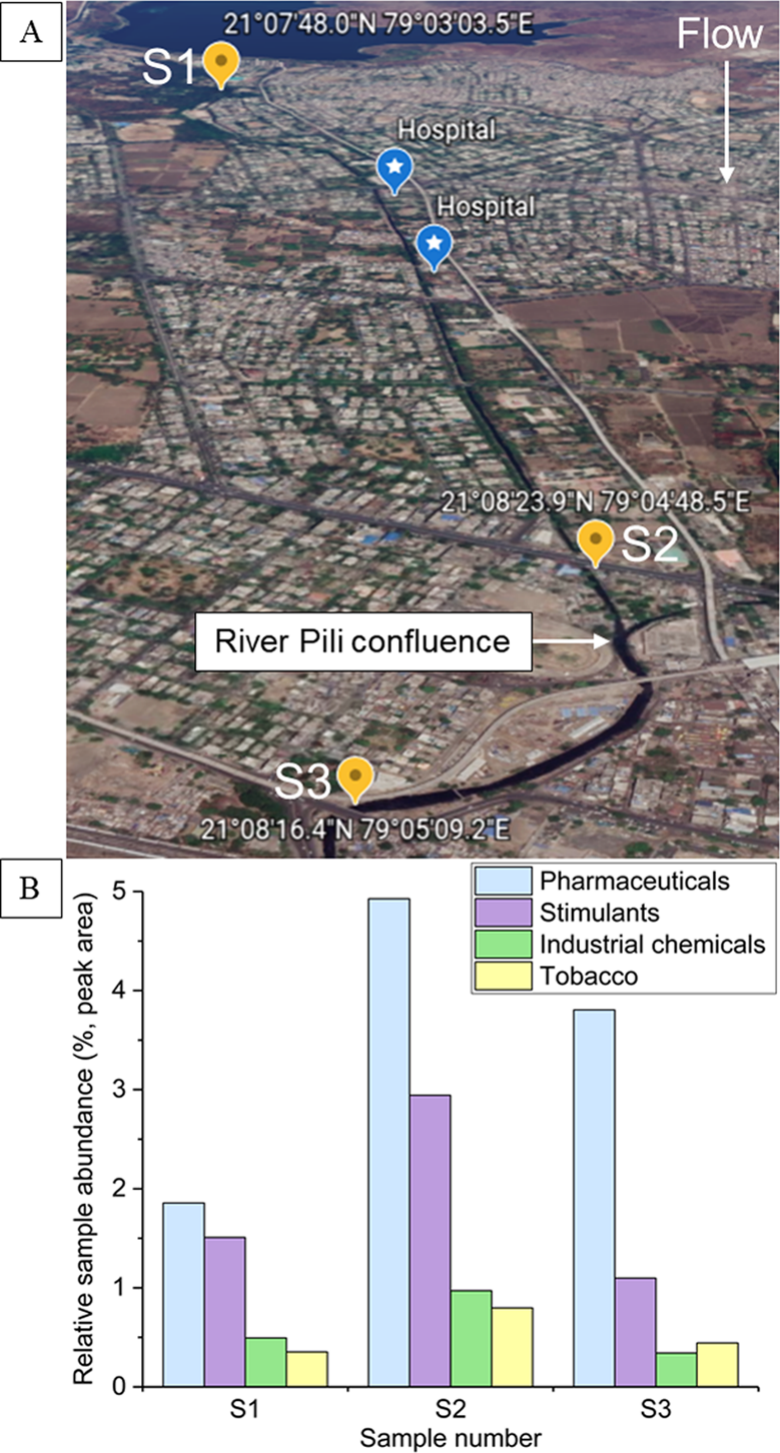
Surface water sampling locations in the River Nag, India (A) and the detected pollutants in each sample with spectral matches >85% confidence (assigned by the commercial mass spectral library, mzCloud) grouped by their potential sources (see Analysis of Surface Waters and Table S11 for further information). The directional flow of the river Nag is shown, along with the confluence of the river Pili. The sample numbers correspond to Table S3. The percentage relative sample abundance was calculated by dividing the measured peak area of each compound by the total sample peak area and multiplying by 100.

Hospital and urban effluents are the main sources of pharmaceuticals, disinfectants, and detergents in surface waters.^51,54^ These pollutants are typically released into the wastewater network, undergoing treatment before being discharged into surface waters, although their removal is known to be inefficient.^49,55^ This is exacerbated in developing countries, where limited facilities can result in the discharge of wastewaters into the environment without prior treatment^56^ The non-targeted method can identify point-source pollution, providing holistic information on compositional changes detected in samples collected from different geographical locations, as shown in Figure 3 for the River Nag, India. Here, it can be observed that the abundance of several pollutant groupings, including tobacco, stimulants, pharmaceuticals, and industrial chemicals, increased downstream of a major urban area (S1 to S2) and then decreased further downstream after dilution from the confluence of a separate less polluted river (Pili river,^57^ S3), following expected trends for potential effluent discharge in the urban area. This was particularly evident for the pharmaceutical grouping, which increased in abundance by a factor of three downstream of two major hospitals, a pollution source previously observed in developing countries.^58^ Nagpur produces over 450 million liters of wastewater per day, but less than one-fifth undergoes treatment before being discharged into the River Nag,^57^ potentially accounting for these observations.

## Discussion and Limitations

Targeted methods, while suited to regulatory activities, provide limited compositional information and rarely capture the heterogeneity of the natural environment. Automated non-targeted screening tools offer considerable advances within the environmental sciences, but these methods (as shown here) are not infallible. It is imperative that non-targeted methods are rigorously tested to provide a fundamental understanding of their potential limitations, especially as their use increases within the environmental sciences. The developed method, at a minimum, provides a level 5 confidence in molecular assignment (i.e., exact mass) as defined by Schymanski et al. (2014). However, for the vast majority of compounds, a level 3 identification confidence is achieved, providing unambiguous molecular formula assignment and MS^2^ data, allowing the compound structure, substituent, or class to be tentatively assigned. The mass spectral libraries provided greater confidence in assignment, including probable structure through MS^2^ library matches (level 2) and confirmed structure via targeted screening using authentic standards (level 1, highest identification confidence). We note that there is also further potential to improve the identification of unknowns in the environmental sciences. Commercial MS^2^ libraries are rarely used in the environmental domain.^59^ We show how the use of the commercial library, mzCloud, can offer advances for non-targeted identification (i.e., rapid identification of probable structure, level 2 confidence^60^), and as this database grows, increased molecular identifications can be anticipated.

One of the main analytical challenges in the analysis of complex matrices is the semiquantitative or quantitative measurement of unknown compounds. Molecular structure can have a considerable impact on ESI efficiencies^61^ and sample extraction recoveries.^62^ These should be recognized as potential limitations. For example, normalized sample abundance exploited here and commonly used in the environmental sciences^63−66^ does not account for different ESI efficiencies. Large differences in ESI efficiencies of individual compounds may distort or disproportionately affect the normalized abundance of the chemical groupings, particularly where few compounds are detected, or where sample compositions vastly differ. Moreover, to determine the recovery efficiency of the sample extraction procedure, the molecular identity of each compound (out of the thousands detected) must be known. Only then can authentic standards be used to accurately quantify recovery efficiencies, assuming that commercial standards are available (a known difficulty in the environmental domain^67^). Internal standards are also plagued by the same challenge, i.e., often not representative of the sheer number of chemically diverse compounds present in environmental matrices. Here, we used common practice sample extraction procedures for PM (e.g., ref (68) and references therein). We did not however investigate the recovery efficiencies of the authentically identified compounds and therefore recommend that in future work, such analysis is performed to quantify any potential losses and provide insight into the quality of the extraction procedure.

To our knowledge, we provide the most rigorously tested automated non-targeted methodology for the compositional analysis of environmental matrices. While the underlying algorithms in the framework can be further improved (i.e., detection of sodium adducts and use of accurate mass resolution for chromatographic peak detection), the approaches shown offer substantial advances from traditional targeted approaches, providing a more complete assessment of sample compositions while significantly increasing throughput. Data can be analyzed unsupervised on a desktop computer in a few hours, in comparison to months of continuous manual processing.

## Supporting Information Available

UPLC-MS and data program method, discussion on the removal of system artifacts, sodium adduct detection, and software notes (PDF)
