# Supplementary material for: An Automated Methodology for Non-targeted Compositional Analysis of Small Molecules in High Complexity Environmental Matrices Using Coupled Ultra Performance Liquid Chromatography Orbitrap Mass Spectrometry
Source: Environ Sci Technol. 2021 May 18;55(11):7365–75. doi: 10.1021/acs.est.0c08208 (PMC8277131; doi:10.1021/acs.est.0c08208)
Supplement: Supplementary file 1 — es0c08208_si_001.pdf [file es0c08208_si_001.pdf]

An automated methodology for non-targeted compositional analysis of small molecules in high complexity environmental matrices using coupled ultra-performance liquid chromatography Orbitrap mass spectrometry

Kelly L. Pereira[1], Martyn W. Ward[1], John L. Wilkinson[2], Jonathan Brett Sallach [2], Daniel J. Bryant[1], William J. Dixon[1], Jacqueline F. Hamilton[1], Alastair C. Lewis[1]*

[1]Wolfson Atmospheric Chemistry Laboratories, Department of Chemistry, University of York, York, YO10 5DD, UK. [2]Department of Environment and Geography, University of York, York, YO10 5NG, UK

[*]Corresponding author; e-mail: kelly.pereira@york.ac.uk, phone: +44 (0)1904 321220

Supplementary Information, Number of Pages: 34

Contents:

1. UPLC-MS Method
2. Data Processing Program
3. Removal of Artefacts
4. Sodium Adduct Detection
5. Software Notes
6. Tables S1 to S11
7. Figures S1 to S10

1. UPLC-MS Method. Compound separation was achieved using a 100 mm × 2.1 mm reverse phase C_{18} polar end-capped column with a 2.6 µm particle size (Accucore aQ, ThermoFisher Scientific). The use of a polar extraction solvent and a reverse-phase C_{18} column for the analysis of organic aerosol is common practice (*e.g.* [1-3]). This combination allows highly oxidized species to be extracted from the sample (often of considerable interest[4]), whilst allowing the separation of less oxidized larger molecular weight compounds such as oligomers, which can represent a major component of organic aerosol[5]. The mobile phase consisted of water with 0.1% (v/v) of formic acid (98% purity, Acros Organics) (A) and methanol (B) (optima LC-MS grade). Gradient elution was used, starting at 90% (A) with a 1-minute post-injection hold, decreasing to 10% (A) at 26 minutes, returning to the starting mobile phase conditions at 28 minutes, followed by a 2-minute hold allowing the re-equilibration of the column. The flow rate was set to 0.3 mL/min. A sample injection volume of 2 µL was used for the analysis of the standards and $PM_{2.5}$ samples. The sample injection volume was increased to 6 µL for the analysis of the surface water samples and corresponding standard calibrations. The sample sequence was run in the following order: solvent blanks, calibration standards, procedural blanks and environmental samples. A quality control standard consisting of the standard mixture at a concentration of 1 ppm was run multiple times throughout the sequence to monitor for instrument sensitivity and drift. Solvent blanks were run at the beginning of the sequence and every ~6 injections, including after the highest concentration standard and more frequently during the analysis of the environmental samples (every 3 injections). The analyses were completed within ~2 days, with an uninterrupted analysis sequence (*i.e.* the analysis of all standard calibrations and environmental samples were performed at the same time). The column temperature was set to 40 °C. Samples were stored in a temperature-controlled autosampler tray during analysis, which was set to 4 °C. Heated electrospray ionization

was used. The capillary and auxiliary gas heater temperatures were set to 320 °C, with a sheath gas flow rate of 70 (arb.) and an auxiliary gas flow rate of 3 (arb.). Spectra were acquired in negative and positive ionization mode with a scan range of mass-to-charge (*m/z*) 85 to 750. Tandem mass spectrometry was performed using higher-energy collision dissociation with a stepped normalized collision energy of 65, 115. The isolation window was set to *m/z* 2.0 with a loop count of 10, selecting the 10 most abundant species for fragmentation in each scan. The chromatographic peak width was set to 6 seconds (full width at half maximum, FWHM) with an apex trigger of 2 to 4 seconds.

2. Data Processing Program. The data processing program requires users to select which data files are 'blanks' (including solvent/instrument and method procedural blanks) and 'samples'. The program will then remove any artefacts detected in the blanks from the sample data, if a sample compound has the following features in common: (i) same detected molecular species (*i.e.* deprotonated, protonated, sodiated) (ii) *m/z* ratio within 2 ppm mass accuracy, (iii) retention time within ±0.1 minutes and, (iv) sample/artefact peak area ratio > 3. Any artefacts detected in the instrument and procedural blanks were removed from the sample data. Further, any compounds which were assigned a molecular formula outside the following tolerances were excluded from the data set: oxygen-to-carbon (O/C) ratio 0.05 to 2 and hydrogen-to-carbon (H/C) ratio of 0.5 to 3. For the surface water samples, the minimum H/C ratio was decreased to 0.33, allowing less oxidized species to be included. The data program calculates the following environmental chemical metrics: H/C ratio, O/C ratio, double bond equivalency (DBE)[6], DBE *vs* carbon (DBE/C)[6], aromaticity index (rAI_{mod})[7] and the average carbon oxidation state ($\overline{\mathrm{O}}\mathrm{Sc}$)[8]. In addition to these calculations, the data program outputs several compositional groupings to allow for the rapid comparison of sample compositions, see below for further information.

For the analysis of PM, all detected compounds in each sample were grouped by their elemental composition and the number of carbon atoms in each molecular formula. The elemental groupings included compounds containing CHO, CHON, CHONS and CHOS. Carbon number groupings consisted of C_2 and C_3, C_4 and C_5, C_6 to C_8, C_9 to C_{10}, C_{11} to C_{15}, C_{16} to C_{20} and C_{21} >. The carbon number groupings were selected to represent potential compound classes and/or sources of abundant species in ambient air. For example, the C_4 and C_5 grouping may be indicative of isoprene oxidation products, C_6 to C_8 grouping of aromatic species, C_9 and C_{10} grouping of monoterpene oxidation products and the C_{11} to C_{15} grouping may include potential sesquiterpene oxidation products. The peak areas of each compound were normalized to the total peak area in each sample, allowing the relative abundance of the chemical groupings between samples to be compared.

For the surface water samples, the data program was designed to output the chemical composition using the commonly reported literature groupings, including: (i) aromatic compounds ($0.66 > AI_{mod} > 0.5$)[6], (ii) polycyclic aromatics ($AI_{mod} > 0.66$ and $C < 14$), (iii) combustion derived black carbon polycyclic aromatics ($AI_{mod} > 0.66$ and $C > 15$), (iv) unsaturated aliphatic compounds containing nitrogen, including peptides ($2 > H/C > 1.5$ and N atom number = 0), (v) unsaturated aliphatic compounds ($2 \geq H/C$ ratio >1.5 and N atom number = 0), (vi) highly unsaturated compounds, including lignin degradation products[9] and carboxyl-rich alicyclic molecules[10] ($AI_{mod} < 0.5$ and H/C ratio < 1.5), (vii) saturated compounds, including lipids (H/C ratio > 2 and O/C ratio < 0.9) and (viii) saturated compounds, including carbohydrates (H/C ratio > 2 and O/C ratio > 0.9). To aid in the compositional interpretation of the surface water samples, any compounds which were identified by the commercial MS^2 library (*i.e.* mzCloud) with spectral matches >85%

confidence were manually grouped into potential pollutant source categories. These categories were selected based on the normalized sample abundance of the tentatively identified compounds and included: (i) industrial chemicals, (ii) pharmaceuticals, (iii) stimulants, (iv) fatty acids, (v) tobacco-related, (vi) plant hormones and (vii) human and animal waste (*e.g.* sewage). N,N-diethyl-meta-toluamide (*i.e.* DEET) was included as a separate group due to its abundance in several surface water samples. Any compounds which could not be described by the above categories were included in a separate grouping labelled 'not assigned'.

The data program outputs into an excel-readable format, allowing users who are not experienced in Python to use the method. Removed system artefacts are recorded in a separate excel sheet to allow the data to be checked. A trial license of Compound Discoverer can be obtained from the manufacturer's website (https://thermo.flexnetoperations.com/), to allow users to test the developed method for their application.

3. Removal of System Artefacts. Artefacts introduced into the sample data from the instrumentation and/or extraction procedure (*i.e.* background compounds) can be performed in Compound Discoverer *via* the 'group unknown compounds' node, see Figure S1. This node groups compounds in all data files with the same *m/z* ratio (within a specified mass accuracy) and set retention time window. The grouping of unknown compounds, particularly within highly complex sample matrices however, results in isomeric species with similar retention times being incorrectly reported as the same compound. To overcome this, the retention time window in the group unknown compounds node was set to 0 minutes, preventing the grouping of any compounds unless detected at the same retention time. This restriction however, prevented the software from removing background compounds from the sample data. To overcome this, the developed data-

processing program was used. The data processing program uses a more restrictive criteria to identify system artefacts in the sample data, minimizing the number of sample components which may be determined to be background compounds (see section ‘data processing program’ for further information).

4. Sodium Adduct Detection. The method initially searches for protonated molecular species in positive ionization mode. If detected, the software then searches for sodium adduct. Consequently, the software cannot detect any compounds which are exclusively observed as $[M+Na]^+$ in positive ionization mode. There were 11 standards which were exclusively detected as $[M+Na]^+$ species (determined *via* manual analysis). The method was unable to detect the chromatographic peaks for 9 out of the 11 compounds. The other 2 compounds, hexanedioic acid and cyclohexane-1,4-dicarboxylic acid, were observed as $[M+H]^+$ species *via* manual analysis but were excluded from the data set as the chromatographic peaks were determined to be <LOD. The non-targeted method integrates chromatographic peaks using a filtered extracted ion chromatogram trace, which smooths the chromatographic peak by summing the centroids found for each data point. This smoothing algorithm was not used for manual analysis. Chromatographic peaks were instead manually integrated, allowing the integration capabilities of the software to be evaluated. The use of the two different chromatographic integration techniques, resulted in a slight variation in the cut-off point for the applied 3 × S/N ratio between the two methods. This variation resulted in the detection of protonated hexanedioic acid and cyclohexane-1,4-dicarboxylic acid using the non-targeted method, subsequently, resulting in the detection of the sodium adducts. The $[M+Na]^+$ chromatographic peaks of camphorsulfonic acid and 2-methyl-4-nitrophenol were however, not detected by the non-targeted method, despite the detection of the protonated adducts. The $[M+Na]^+$

chromatographic peaks of camphorsulfonic acid and 2-methyl-4-nitrophenol were clearly visible in the chromatograms, with an S/N ratio of 243 and 25, respectively (determined *via* manual analysis); it is unclear why the software did not detect these species.

5. Software Notes. In the initial design of our method, the S/N ratio threshold was set to 3 in the select spectra and detect unknown compounds node (see Figure S1). Interestingly, it was found that restricting the S/N threshold to 3 in the select spectra node, increased the number of low concentration species which were incorrectly determined to be below the LOD. It is therefore recommended that the S/N threshold is set to 0 in the select spectra node and 3 in the detect unknown compounds node, effectively bypassing this initial restriction.

Table S1 - Compound names, manufacturer and purity of the standards.

**Compound name**	**Manufacturer**	**Purity**	**CAS number**	**MW**	**MF**
cyclohex-2-en-1-one	a	95.0	930-68-7	96.13	C_6H_8O
furan-2,5-dione	B	99.0	108-31-6	98.06	$C_4H_2O_3$
propanedioic acid	a	99.0	141-82-2	104.06	$C_3H_4O_4$
(Z)-but-2-enedioic acid	B	99.0	110-16-7	116.07	$C_4H_4O_4$
4-oxopentanoic acid	B	97.0	123-76-2	116.12	$C_5H_8O_3$
butanedioic acid	a	99.0	110-15-6	118.09	$C_4H_6O_4$
2-hydroxy-3-methylbutanoic acid	A	99.0	4026-18-0	118.13	$C_5H_{10}O_3$
2-methylbenzaldehyde	a	97.0	529-20-4	120.15	C_8H_8O
4-methylbenzaldehyde	a	97.0	104-87-0	120.15	C_8H_8O
benzoic acid	a	99.5	65-85-0	122.12	$C_7H_6O_2$
4-methylbenzene-1,2-diol	a	95.0	452-86-8	124.14	$C_7H_8O_2$
3-methylbenzene-1,2-diol	a	98.0	488-17-5	124.14	$C_7H_8O_2$
octan-2-one	B	97.0	111-13-7	128.21	$C_8H_{16}O$
(Z)-2-methylbut-2-enedioic acid	a	98.0	498-23-7	130.10	$C_5H_6O_4$
Pentanedioic acid	a	99.0	110-94-1	132.11	$C_5H_8O_4$
dimethyl propanedioate	C	99.0	108-59-8	132.11	$C_5H_8O_4$
2-ethoxyethyl acetate	C	99.0	111-15-9	132.16	$C_6H_{12}O_3$
2-hydroxyhexanoic acid	C	95.0	6064-63-7	132.16	$C_6H_{12}O_3$
2,5-dimethylbenzaldehyde	a	99.0	5779-94-2	134.18	$C_9H_{10}O$
4-methoxybenzaldehyde	a	98.0	123-11-5	136.15	$C_8H_8O_2$
4-methylbenzoic acid	C	98.0	99-94-5	136.15	$C_8H_8O_2$
3-methylbenzoic acid	a	99.0	99-04-7	136.15	$C_8H_8O_2$
2-hydroxybenzoic acid	a	99.0	69-72-7	138.12	$C_7H_6O_3$
4-nitrophenol	A	99.0	100-02-7	139.11	$C_6H_5NO_3$
3-nitrophenol	C	99.0	554-84-7	139.11	$C_6H_5NO_3$
hexanedioic acid	a	99.0	124-04-9	146.14	$C_6H_{10}O_4$
(E)-3-phenylprop-2-enoic acid	C	99.0	140-10-3	148.16	$C_9H_8O_2$
2-formylbenzoic acid	a	97.0	119-67-5	150.13	$C_8H_6O_3$
4-methoxybenzoic acid	a	99.0	100-09-4	152.15	$C_8H_8O_3$
2-methyl-5-nitrophenol	E	98.0	5428-54-6	153.14	$C_7H_7NO_3$
4-methyl-3-nitrophenol	C	98.0	2042-14-0	153.14	$C_7H_7NO_3$
4-methyl-2-nitrophenol	a	97.0	119-33-5	153.14	$C_7H_7NO_3$
2-methyl-4-nitrophenol	a	97.0	99-53-6	153.14	$C_7H_7NO_3$
5-methyl-2-nitrophenol	C	97.0	700-38-9	153.14	$C_7H_7NO_3$
2-methyl-3-nitrophenol	E	98.0	5460-31-1	153.14	$C_7H_7NO_3$

MW = molecular weight. MF = molecular formula. a = Sigma Aldrich, UK; b = Honeywell Fluka, UK; c = Fisher Scientific, UK; e = Fluorochem, UK; f = Tokyo Chemical Industry (TCI), UK.

Table S1 (continued) - Compound names, manufacturer and purity of the standards.

Compound name	Manufacturer	Purity	CAS number	MW	MF
3-methyl-4-nitrophenol	f	98.0	2581-34-2	153.14	$C_7H_7NO_3$
3-methyl-2-nitrophenol	e	95.0	4920-77-8	153.14	$C_7H_7NO_3$
2,5-dihydroxybenzoic acid	c	99.0	490-79-9	154.12	$C_7H_6O_4$
2,6-dimethoxyphenol	a	99.0	91-10-1	154.16	$C_8H_{10}O_3$
(3R)-3,7-dimethyloct-6-enal	a	95.0	2385-77-5	154.25	$C_{10}H_{18}O$
2-nitrobenzene-1,3-diol	a	98.0	601-89-8	155.11	$C_6H_5NO_4$
4-nitrobenzene-1,2-diol	a	97.0	3316-09-4	155.11	$C_6H_5NO_4$
nonanoic acid	a	97.0	112-05-0	158.24	$C_9H_{18}O_2$
heptanedioic acid	c	98.0	111-16-0	160.17	$C_7H_{12}O_4$
levoglucosan	a	99.0	498-07-7	162.14	$C_6H_{10}O_5$
1,2-benzenedioic acid	a	99.5	88-99-3	166.13	$C_8H_6O_4$
2,6-dimethyl-4-nitrophenol	a	98.0	2423-71-4	167.16	$C_8H_9NO_3$
1,3,5-trimethoxybenzene	a	99.0	621-23-8	168.19	$C_9H_{12}O_3$
2-methoxy-4-nitrophenol	a	97.0	3251-56-7	169.13	$C_7H_7NO_4$
cyclohexane-1,4-dicarboxylic acid	c	99.0	1076-97-7	172.18	$C_8H_{12}O_4$
octanedioic acid	c	99.0	505-48-6	174.19	C_8H_{14}O4
2-hydroxy-5-nitrobenzoic acid	a	99.0	96-97-9	183.12	$C_7H_5NO_5$
2,4-dinitrophenol	a	97.0	51-28-5	184.11	$C_6H_4N_2O_5$
cis-pinonic acid	a	98.0	61826-55-9	184.23	$C_{10}H_{16}O_3$
nonanedioic acid	c	98.0	123-99-9	188.22	$C_9H_{16}O_4$
(4-formyl-2-methoxyphenyl) acetate	a	97.0	881-68-5	194.18	$C_{10}H_{10}O_4$
naphthalene-2,3-dicarboxylic acid	a	95.0	2169-87-1	216.19	$C_{12}H_8O_4$
2,3-diacetyloxypropyl acetate	c	99.0	102-76-1	218.20	$C_9H_{14}O_6$
β-caryophyllene epoxide	a	99.0	1139-30-6	220.35	$C_{15}H_{24}O$
1s-(+)-camphorsulfonic acid	a	99.0	3144-16-9	232.30	$C_{10}H_{16}O_4S$

MW = molecular weight. MF = molecular formula. a = Sigma Aldrich, UK; b = Honeywell Fluka, UK; c = Fisher Scientific, UK; e = Fluorochem, UK; f = Tokyo Chemical Industry (TCI), UK.

Table S2 – Particulate matter sample sampling dates, times and the volume of air sampled during sample collection.

Winter				
Sample number	94	96	98	100
Sampling start date (DD:MM:YY)	29/11/16	29/11/16	30/11/16	30/11/16
Sampling end date (DD:MM:YY)	29/11/16	30/11/16	30/11/16	01/12/16
Sampling start time (HH:MM)	11:35	17:38	11:33	17:35
Sampling end time (HH:MM)	14:31	08:29	14:26	08:30
Sampling duration (HH:MM)	02:56	14:51	02:53	14:55
Sampling duration (min)	176	891	173	895
Volume of air sampled (m^3)	234.7	1188.0	230.7	1193.3
Summer				
Sample number	261*	264*	271*	274*
Sampling start date (DD:MM:YY)	17/06/17	17/06/17	18/06/17	18/06/17
Sampling end date (DD:MM:YY)	17/06/17	18/06/17	18/06/17	19/06/17
Sampling start time (HH:MM)	14:28	18:30	14:36	17:30
Sampling end time (HH:MM)	15:23	08:34	15:24	08:36
Sampling duration (HH:MM)	00:55	14:04	00:48	15:06
Sampling duration (min)	55	844	48	906
Volume of air sampled (m^3)	73.3	1125.3	64.0	1208.0

*Same samples as those analyzed in Bryant et al. 2019.

Table S3 – Surface water sample descriptions and collection locations.

Sample ID	Sample description	Country	City	River	Co-ordinates (Lat. Long.) (if known)
S1	Industrial effluent	Sri Lanka	Colombo	-	-
S2	Hong Kong (sewage and building construction influence)	China	Hong Kong	Kai Tak	22° 19’ 45.8” N, 114° 11’ 53.9” E
S3	Wastewater treatment plant effluent, WWTP	Sri Lanka	Colombo	-	-
S4	Guangzhou (pharmaceuticals, agricultural and sewage influence)	China	Guangzhou	Zhujiang	23° 07’ 23.2” N, 113° 12’ 33.8” E
S5	Nagpur (upstream of two major hospitals)	India	Nagpur	Nag	21° 07' 48.0” N, 79° 03' 03.5” E
S6	Nagpur (downstream of two major hospitals)	India	Nagpur	Nag	21° 08' 23.9” N, 79° 04' 48.5” E
S7	Nagpur (downstream of S6, post River Pili confluence)	India	Nagpur	Nag	21° 08' 16.4” N, 79° 05' 09.2” E

Table S4 – Compound Discoverer library used for the detection of ESI artefacts.

Adduct	Adduct Mass (Da)	Charge
M-H-H_2O	-19.01784	-1
M+H-H_2O	-17.00329	1
M+H-NH_3	-16.01927	1
M-2H	-2.01455	-2
M-H	-1.00728	-1
2M-H	-1.00728	-1
M+H	1.00728	1
2M+H	1.00728	1
M+2H	2.01455	2
M+3H	3.02183	3
M+NH_4	18.03383	1
2M+NH_4	18.03383	1
M+H+NH_4	19.0411	2
M+Na	22.98922	1
2M+Na	22.98922	1
M+H+Na	23.9965	2
M+H+MeOH	33.03349	1
M+Cl	34.9694	-1
M-2H+K	36.94861	-1
M+K	38.96316	1
2M+K	38.96316	1
M+H+K	39.97044	2
M+H+ACN	42.03383	1
2M+H+ACN	42.03383	1
M+2H+ACN	43.0411	2
M-H+FA	44.9982	-1
2M-H+FA	44.9982	-1
M-H+HAc	59.01385	-1
2M-H+HAc	59.01385	-1
M+Na+ACN	64.01577	1
2M+Na+ACN	64.01577	1
M+H+DMSO	79.02121	1
M-H+TFA	112.98559	-1

Table S5– Retention time and the type of molecular species detected for each standard at a concentration of 1 ppm determined *via* manual analysis.

Compound name	Retention time (min)	$(M-H)^-$	$(M+H)^+$	$(M+Na)^+$
levoglucosan	0.75			✓
propanedioic acid	0.83	✓		
(Z)-but-2-enedioic acid	0.86	✓		
butanedioic acid	0.99	✓		✓
(Z)-2-methylbut-2-enedioic acid	1.22	✓		
4-oxopentanoic acid	1.27	✓		✓
pentanedioic acid	1.33	✓		✓
furan-2,5-dione	1.37	✓	✓	
2-hydroxy-3-methylbutanoic acid	2.40	✓		✓
hexanedioic acid	2.41	✓		✓
dimethyl propanedioate	2.68		✓	✓
2,5-dihydroxybenzoic acid	3.04	✓	✓	
2-formylbenzoic acid	3.75	✓	✓	✓
1,2-benzenedioic acid	3.80	✓	✓	✓
cyclohex-2-en-1-one	4.13		✓	
1s-(+)-camphorsulfonic acid	4.32	✓	✓	✓
4-nitrobenzene-1,2-diol	4.61	✓		
heptanedioic acid	4.74	✓		✓
4-methylbenzene-1,2-diol	4.85	✓		
2-ethoxyethyl acetate	5.24			✓
3-methylbenzene-1,2-diol	5.33	✓		
2-hydroxyhexanoic acid	5.57	✓		✓
2-nitrobenzene-1,3-diol	5.75	✓		
cyclohexane-1,4-dicarboxylic acid	5.88	✓		✓
4-nitrophenol	6.54	✓		
2-hydroxy-5-nitrobenzoic acid	6.57	✓		
3-nitrophenol	7.02	✓		
2,3-diacetyloxypropyl acetate	7.23			✓
2,6-dimethoxyphenol	7.29		✓	✓
octanedioic acid	7.67	✓	✓	✓
2,4-dinitrophenol	7.72	✓		
2-methoxy-4-nitrophenol	7.90	✓	✓	✓
benzoic acid	7.93	✓		
2-hydroxybenzoic acid	7.93	✓		
cis-pinonic acid	8.27	✓	✓	✓

Table S5 (continued) – Retention time and the type of molecular species detected for each standard at a concentration of 1 ppm determine *via* manual analysis.

**Compound name**	**Retention time (min)**	**(M-H)$^-$**	**(M+H)$^+$**	**(M+Na)$^+$**
4-methoxybenzoic acid	9.28	✓	✓	
4-methoxybenzaldehyde	9.29		✓	
(4-formyl-2-methoxyphenyl) acetate	9.36		✓	✓
3-methyl-4-nitrophenol	9.59	✓	✓	
2-methyl-3-nitrophenol	9.99	✓		
3-methyl-2-nitrophenol	10.08	✓		
4-methyl-3-nitrophenol	10.21	✓		
nonanedioic acid	10.50	✓	✓	✓
2-methyl-4-nitrophenol	10.69	✓	✓	✓
naphthalene-2,3-dicarboxylic acid	10.84	✓	✓	✓
2-methyl-5-nitrophenol	11.28	✓		
4-methylbenzaldehyde	11.33		✓	
2-methylbenzaldehyde	11.38		✓	
4-methylbenzoic acid	11.57	✓	✓	
3-methylbenzoic acid	11.62	✓	✓	
(E)-3-phenylprop-2-enoic acid	11.76	✓		
2,6-dimethyl-4-nitrophenol	12.93	✓	✓	✓
4-methyl-2-nitrophenol	13.16	✓		
5-methyl-2-nitrophenol	13.17	✓		
1,3,5-trimethoxybenzene	13.29		✓	
2,5-dimethylbenzaldehyde	14.88		✓	
octan-2-one	16.28		✓	
(3R)-3,7-dimethyloct-6-enal	18.89		✓	
nonanoic acid	19.31	✓		
β-caryophyllene epoxide	22.43		✓	✓

Table S6 – Number of compounds detected in the particulate matter samples in negative and positive ionization mode.

Winter	Number of detected compounds			
	Sample number	Negative ionization mode	Positive ionization mode	Total
	94	4118	4154	7852
	96	4887	4768	7157
	98	3508	3649	9655
	100	3939	3913	8272
Summer	261	1887	1390	3277
	264	4947	4331	9278
	271	2453	1949	4402
	274	4415	4281	8696

Sample numbers correspond to Table S2.

Table S7 – Concentrations (in air) of the quantified compounds in the $PM_{2.5}$ samples collected in the summer season.

Compound	MW	MF	Retention time (t_R)	Molecular species	Sample 261 (ng/m^3)	Sample 264 (ng/m^3)	Sample 271 (ng/m^3)	Sample 274 (ng/m^3)
propanedioic acid	104.06	$C_3H_4O_4$	0.83	$(M-H)^-$	-	24.89	-	-
(Z)-but-2-enedioic acid	116.07	$C_4H_4O_4$	0.86	$(M-H)^-$	24.53	4.92	48.29	2.18
butanedioic acid	118.09	$C_4H_6O_4$	0.97	$(M-H)^-$	28.80	8.00	44.53	2.70
(Z)-2-methylbut-2-enedioic acid	130.1	$C_5H_6O_4$	1.22	$(M-H)^-$	20.98	3.37	43.10	1.88
4-oxopentanoic acid	116.12	$C_5H_8O_3$	1.27	$(M-H)^-$	168.57	**	352.07	-
pentanedioic acid	132.11	$C_5H_8O_4$	1.33	$(M-H)^-$	9.51	3.18	17.57	1.65
2-hydroxy-3-methylbutanoic acid	118.13	$C_5H_{10}O_3$	2.39	$(M-H)^-$	-	0.15	*	0.05
hexanedioic acid	146.14	$C_6H_{10}O_4$	2.3	$(M-H)^-$	5.22	3.86	10.53	2.28
2-formylbenzoic acid	150.13	$C_8H_6O_3$	3.75	$(M-H)^-$	21.40	2.17	29.35	0.56
1,2-benzenedioic acid	166.13	$C_8H_6O_4$	3.55	$(M-H)^-$	61.46	20.47	83.30	17.50
4-nitrobenzene-1,2-diol	155.11	$C_6H_5NO_4$	4.44	$(M-H)^-$	*	1.50	*	0.69
heptanedioic acid	160.17	$C_7H_{12}O_4$	4.55	$(M-H)^-$	30.05	0.60	1.87	0.96
2-hydroxyhexanoic acid	132.16	$C_6H_{12}O_3$	5.57	$(M-H)^-$	*	0.02	0.05	0.01
4-nitrophenol	139.11	$C_6H_5NO_3$	6.22	$(M-H)^-$	9.30	1.06	21.25	0.39
2-hydroxy-5-nitrobenzoic acid	183.12	$C_7H_5NO_5$	6.28	$(M-H)^-$	0.83	**	1.55	**
2,4-dinitrophenol	184.11	$C_6H_4N_2O_5$	7.39	$(M-H)^-$	0.51	0.14	0.66	0.02
octanedioic acid	174.19	$C_8H_{14}O_4$	7.43	$(M-H)^-$	0.84	2.87	1.57	1.74
2-methoxy-4-nitrophenol	169.13	$C_7H_7NO_4$	7.64	$(M-H)^-$	-	-	*	-
2-hydroxybenzoic acid	138.12	$C_7H_6O_3$	7.52	$(M-H)^-$	0.00	0.67	1.91	0.23
3-methyl-4-nitrophenol	153.14	$C_7H_7NO_3$	9.24	$(M-H)^-$	0.54	0.02	1.33	0.01
nonanedioic acid	188.22	$C_9H_{16}O_4$	10.48	$(M-H)^-$	0.71	**	2.30	6.16
2-methyl-4-nitrophenol	153.14	$C_7H_7NO_3$	10.28	$(M-H)^-$	3.00	0.08	8.56	0.02
naphthalene-2,3-dicarboxylic acid	216.19	$C_{12}H_8O_4$	10.84	$(M-H)^-$	-	-	-	0.02
2,6-dimethyl-4-nitrophenol	167.16	$C_8H_9NO_3$	12.56	$(M-H)^-$	0.97	0.04	1.38	0.003
cis-pinonic acid	184.23	$C_{10}H_{16}O_3$	8.27	$(M+H)^+$	*	3.37	*	2.82
dimethyl propanedioate	132.11	$C_5H_8O_4$	2.68	$(M+H)^+$	-	0.39	-	0.07
2,5-dimethylbenzaldehyde	134.18	$C_9H_{10}O$	14.88	$(M+H)^+$	*	-	*	-
1,3,5-trimethoxybenzene	168.19	$C_9H_{12}O_3$	13.29	$(M+H)^+$	-	0.03	-	-
			Number of compounds quantified		22	25	24	23
			Total OA mass quantified (µg/m^3)		0.39	0.08	0.67	0.04
			Average $PM_{2.5}$ mass during sampling (µg/m^3)		95	83	61	33
			Amount of OA mass quantified (%)		0.41	0.10	1.10	0.13

MW = molecular weight. MF = molecular formula. - = Not detected, * = Below linear calibration range, ** = Above linear calibration range.

Table S8 – Concentrations (in air) of the quantified compounds in the $PM_{2.5}$ samples collected in the winter season.

Compound	MW	MF	Retention time (t_R)	Molecular species	Sample 94 (ng/m^3)	Sample 96 (ng/m^3)	Sample 98 (ng/m^3)	Sample 100 (ng/m^3)
(Z)-but-2-enedioic acid	116.07	$C_4H_4O_4$	0.86	$(M-H)^-$	8.47	2.58	10.37	1.71
butanedioic acid	118.09	$C_4H_6O_4$	0.97	$(M-H)^-$	5.98	2.30	5.01	1.49
(Z)-2-methylbut-2-enedioic acid	130.1	$C_5H_6O_4$	1.22	$(M-H)^-$	8.99	3.30	8.91	1.12
4-oxopentanoic acid	116.12	$C_5H_8O_3$	1.27	$(M-H)^-$	72.22	-	51.69	14.58
2-hydroxy-3-methylbutanoic acid	118.13	$C_5H_{10}O_3$	2.39	$(M-H)^-$	0.20	0.10	0.15	0.06
hexanedioic acid	146.14	$C_6H_{10}O_4$	2.30	$(M-H)^-$	4.10	1.70	4.64	0.60
2,5-dihydroxybenzoic acid	154.12	$C_7H_6O_4$	2.90	$(M-H)^-$	0.35	-	0.38	-
2-formylbenzoic acid	150.13	$C_8H_6O_3$	3.75	$(M-H)^-$	**	**	**	2.75
1,2-benzenedioic acid	166.13	$C_8H_6O_4$	3.55	$(M-H)^-$	57.49	22.15	51.31	2.07
4-nitrobenzene-1,2-diol	155.11	$C_6H_5NO_4$	4.44	$(M-H)^-$	**	**	**	0.84
heptanedioic acid	160.17	$C_7H_{12}O_4$	4.55	$(M-H)^-$	1.52	0.77	1.34	0.23
4-methylbenzene-1,2-diol	124.14	$C_7H_8O_2$	4.59	$(M-H)^-$	0.07	0.27	0.14	-
3-methylbenzene-1,2-diol	124.14	$C_7H_8O_2$	5.06	$(M-H)^-$	0.32	0.42	0.09	-
4-nitrophenol	139.11	$C_6H_5NO_3$	6.22	$(M-H)^-$	**	**	16.66	**
2-hydroxy-5-nitrobenzoic acid	183.12	$C_7H_5NO_5$	6.28	$(M-H)^-$	2.85	**	2.92	0.10
2,4-dinitrophenol	184.11	$C_6H_4N_2O_5$	7.39	$(M-H)^-$	5.90	**	1.54	0.19
octanedioic acid	174.19	$C_8H_{14}O_4$	7.43	$(M-H)^-$	2.00	1.12	2.19	0.40
2-methoxy-4-nitrophenol	169.13	$C_7H_7NO_4$	7.64	$(M-H)^-$	3.07	-	-	0.21
2-hydroxybenzoic acid	138.12	$C_7H_6O_3$	7.52	$(M-H)^-$	8.13	**	5.78	0.94
3-methyl-4-nitrophenol	153.14	$C_7H_7NO_3$	9.24	$(M-H)^-$	**	**	**	**
nonanedioic acid	188.22	$C_9H_{16}O_4$	10.48	$(M-H)^-$	8.67	3.44	8.31	2.59
2-methyl-4-nitrophenol	153.14	$C_7H_7NO_3$	10.28	$(M-H)^-$	**	**	3.14	**
2,6-dimethyl-4-nitrophenol	167.16	$C_8H_9NO_3$	12.56	$(M-H)^-$	5.89	**	3.87	**
nonanoic acid	158.24	$C_9H_{18}O_2$	19.31	$(M-H)^-$	6.29	-	*	-
cis-pinonic acid	184.23	$C_{10}H_{16}O_3$	8.27	$(M+H)^+$	*	-	*	*
4-methoxybenzoic acid	152.15	$C_8H_8O_3$	9.29	$(M+H)^+$	-	-	-	*
2,6-dimethoxyphenol	154.16	$C_8H_{10}O_3$	7.29	$(M+H)^+$	-	-	-	-
		Number of detected compounds			25	20	24	22
		Total OA mass quantified (μg/m^3)			0.20	0.04	0.18	0.03
		Average $PM_{2.5}$ mass during sampling (μg/m^3)			141	111	129	7
		Amount of OA mass quantified (%)			0.14	0.03	0.14	0.42

MW = molecular weight. MF = molecular formula. - = Not detected, * = Below linear calibration range, ** = Above linear calibration range.

Table S9 – Number of compounds detected in the surface water samples in negative and positive ionization mode.

Sample ID	Sample description	Number of detected compounds		
		Negative mode	Positive mode	Total
S1	Colombo (industrial effluent)	3972	5193	9165
S2	Hong Kong (sewage and building construction influence)	434	2387	2821
S3	Colombo, (wastewater treatment effluent)	2447	1483	3930
S4	Guangzhou (pharmaceuticals, agricultural and sewage influence)	1200	1746	2946
S5	Nagpur (upstream of two major hospitals)	357	1643	2000
S6	Nagpur (downstream of two major hospitals)	2768	3041	5809
S7	Nagpur (downstream of S6, post river Pili confluence)	79	1424	1503

Sample numbers correspond to Table S2.

Table S10 – Concentrations of the quantified compounds in the surface water samples in µg L^{-1}.

Compound	MW	MF	Retention time (t_R)	Molecular species	S1	S2	S3	S4	S5	S6	S7
hexanedioic acid	146.14	$C_6H_{10}O_4$	2.3	$(M-H)^-$	[a]	-	[a]	[a]	[a]	-	-
4-nitrobenzene-1,2-diol	155.11	$C_6H_5NO_4$	4.44	$(M-H)^-$	-	-	-	0.71	-	-	-
heptanedioic acid	160.17	$C_7H_{12}O_4$	4.55	$(M-H)^-$	-	-	-	[a]	-	-	-
4-nitrophenol	139.11	$C_6H_5NO_3$	6.22	$(M-H)^-$	0.26	-	0.08	0.15	*	*	*
octanedioic acid	174.19	$C_8H_{14}O_4$	7.43	$(M-H)^-$	0.11	0.18	0.18	0.49	0.49	0.47	0.34
2-hydroxybenzoic acid	138.12	$C_7H_6O_3$	7.52	$(M-H)^-$	0.07	-	0.07	-	0.10	0.05	0.03
benzoic acid	122.12	$C_7H_6O_2$	7.93	$(M-H)^-$	-	*	0.68	13.15	10.42	14.10	15.67
3-methyl-4-nitrophenol	153.14	$C_7H_7NO_3$	9.24	$(M-H)^-$	-	-	-	*	*	*	*
2-methyl-4-nitrophenol	153.14	$C_7H_7NO_3$	10.28	$(M-H)^-$	*	-	-	0.07	*	*	*
nonanedioic acid	188.22	$C_9H_{16}O_4$	10.48	$(M-H)^-$	0.16	0.70	0.39	0.56	0.66	0.60	0.36
2,6-dimethyl-4-nitrophenol	167.16	$C_8H_9NO_3$	12.56	$(M-H)^-$	*	-	-	0.03	*	*	*
nonanoic acid	158.24	$C_9H_{18}O_2$	19.31	$(M-H)^-$	24.97	21.87	24.99	6.67	*	6.95	6.21
(3R)-3,7-dimethyloct-6-enal	154.25	$C_{10}H_{18}O$	18.89	$(M+H)^+$	-	*	-	-	-	-	-
1,2-benzenedioic acid	166.13	$C_8H_6O_4$	3.78	$(M+Na)^+$	-	-	-	-	[a]	-	-
	Total concentration quantified (ppb)				25.57	22.75	26.38	21.84	11.67	22.17	22.61

MW = molecular weight. MF = molecular formula. - = Not detected, S1 – S7 is the sample identifier, see Table S3.

[*] = Below linear calibration range, [a] = Poor chromatographic peak shape prevented quantification.

Table S11 – Compounds names, source categories and relative sample abundances of the compounds tentatively identified in the surface water samples using the commercial mass spectral library, mzCloud.

Sample ID	MF	MW	t_R	Name	Category	mzCloud match (%)	Normalized peak area*
S1	C_7H_5NOS	151.0	6.4	1,2-benzisothiazolin-3-one	industrial	94.0	3.06×10^{-3}
	$C_{24}H_{30}O_6$	414.2	18.7	bis(4-ethylbenzylidene)sorbitol	industrial	90.7	4.32×10^{-4}
	$C_{10}H_{13}N_5O_4$	267.1	1.1	adenosine	pharmaceuticals	95.8	5.44×10^{-4}
	$C_{10}H_{16}O$	152.1	19.6	D-(+)-camphor	pharmaceuticals	86.8	1.94×10^{-3}
	$C_{15}H_{23}NO_2$	249.2	4.2	methamphetamine tert-butyl carbamate	pharmaceuticals	85.1	7.17×10^{-4}
	$C_{13}H_{12}N_2O$	212.1	13.1	N,N'-diphenylurea	human/animal waste	94.5	2.42×10^{-3}
	C_9H_7NO	145.1	8.1	2-hydroxyquinoline	not assigned	88.6	9.41×10^{-4}
	C_6H_7NO	109.1	1.0	3-hydroxy-2-methylpyridine	not assigned	87.9	3.46×10^{-4}
	$C2_0H_{30}O_2$	302.2	25.6	abietic acid	not assigned	86.7	2.04×10^{-4}
	$C_{12}H_{17}NO$	191.1	14.5	DEET	DEET	97.6	1.23×10^{-1}
	$C_8H_{10}N_4O_2$	194.1	5.6	caffeine	stimulants	93.4	6.54×10^{-3}
	C_7H_5NOS	151.0	6.4	1,2-benzisothiazolin-3-one	industrial	94.0	3.06×10^{-3}
	$C_{24}H_{30}O_6$	414.2	18.7	bis(4-ethylbenzylidene)sorbitol	industrial	90.7	4.32×10^{-4}
S2	$C_{24}H_{30}O_6$	414.2	18.74	bis(4-ethylbenzylidene)sorbitol	industrial	92.3	5.69×10^{-3}
	$C_{12}H_{27}O_4P$	266.2	21.25	tributyl phosphate	industrial	91.5	2.02×10^{-3}
	$C_6H_{11}NO$	113.1	3.67	caprolactam	industrial	88.3	2.57×10^{-3}
	$C_{14}H_{22}N_2O$	234.2	4.51	lidocaine	pharmaceuticals	96.4	1.51×10^{-3}
	$C_{21}H_{25}ClN_2O_3$	388.2	15.28	cetirizine	pharmaceuticals	92.0	9.47×10^{-4}
	$C_{32}H_{39}NO_4$	501.3	14.67	fexofenadine	pharmaceuticals	91.2	1.17×10^{-3}
	$C_{17}H_{21}NO$	255.2	11.08	diphenhydramine	pharmaceuticals	90.9	1.49×10^{-3}
	$C_{15}H_{25}NO_3$	267.2	7.13	metoprolol	pharmaceuticals	90.8	6.03×10^{-3}
	$C_{17}H_{23}NO$	257.2	6.67	levorphanol	pharmaceuticals	89.8	5.34×10^{-3}
	$C_{16}H_{25}NO_2$	263.2	5.95	o-desmethylvenlafaxine	pharmaceuticals	88.9	2.87×10^{-3}
	$C_{17}H_{27}N_3O_4S$	369.2	5.28	amisulpride	pharmaceuticals	88.4	2.79×10^{-3}
	$C_{15}H_{12}N_2O$	236.1	13.19	carbamazepine	pharmaceuticals	87.6	1.79×10^{-3}
	$C_{15}H2_3N_3 O_4S$	341.1	1.92	sulpiride	pharmaceuticals	87.2	1.13×10^{-3}
	$C_{15}H_{21}N_3 O_3S$	323.1	15.35	gliclazide	pharmaceuticals	86.7	1.56×10^{-3}
	$C_{15}H_{15}NO_2$	241.1	20.91	mefenamic acid	pharmaceuticals	85.6	8.28×10^{-4}
	$C_{13}H_{10}O_2$	198.1	13.26	4-hydroxybenzophenone	industrial	91.8	7.80×10^{-4}
	$C_9H_9N_3O_2$	191.1	3.75	carbendazim	pesticide	86.4	2.87×10^{-3}
	$C_{12}H_{17}NO$	191.1	14.50	DEET	DEET	92.2	1.73×10^{-2}
S3	$C_{24}H_{30}O_6$	414.20	18.727	bis(4-ethylbenzylidene)sorbitol	industrial	92.7	2.85×10^{-3}
	$C_{10}H_{13}N_5O_4$	267.10	1.067	adenosine	pharmaceuticals	96.1	5.34×10^{-3}
	$C_8H_9NO_2$	151.06	1.989	paracetamol	pharmaceuticals	94.1	1.85×10^{-2}
	$C_{32}H_{39}NO_4$	501.29	14.673	fexofenadine	pharmaceuticals	85.7	1.37×10^{-3}
	$C_5H_{13}NO$	103.10	0.723	choline	not assigned	95.9	1.27×10^{-2}
	$C_5H_7NO_3$	129.04	0.909	L-pyroglutamic acid	not assigned	89.7	2.32×10^{-3}
	$C_{12}H_{17}NO$	191.13	14.504	DEET	DEET	91.5	1.17×10^{-1}
	$C_8H_{10}N_4O_2$	194.08	5.592	caffeine	stimulants	88.9	1.93×10^{-2}

Table S11 (continued) - Compounds names, source categories and relative sample abundances of the compounds tentatively identified in the surface water samples using the commercial mass spectral library, mzCloud.

Sample ID	MF	MW	t_R	Name	Category	mzCloud match (%)	Normalized peak area[*]
S4	$C_{24}H_{30}O_6$	414.2	18.74	bis(4-ethylbenzylidene)sorbitol	industrial	92.5	2.64×10^{-3}
	$C_{12}H_{27}O_4P$	266.2	21.26	tributyl phosphate	industrial	89.7	3.74×10^{-3}
	C18H15OP	278.1	16.74	triphenylphosphine oxide	industrial	89.1	7.44×10^{-3}
	$C_6H_{11}NO$	113.1	3.68	caprolactam	industrial	89.0	5.47×10^{-3}
	$C_{11}H_{15}NO_2$	193.1	7.04	1,3-benzodioxolylbutanamine (BDB)	pharmaceuticals	85.0	2.18×10^{-3}
S5	$C_{24}H_{30}O_6$	414.20	18.739	bis(4-ethylbenzylidene)sorbitol	industrial	92.3	2.25×10^{-3}
	$C_6H_{11}NO$	113.08	3.7	caprolactam	industrial	86.4	2.70×10^{-3}
	$C_{10}H_{13}N_5O_4$	267.10	1.048	adenosine	pharmaceuticals	96.1	1.11×10^{-2}
	$C_4H_7N_3O$	113.06	0.844	creatinine	human/animal waste	91.1	1.14×10^{-2}
	$C_{17}H_{19}NO_3$	285.14	18.308	piperine[†]	not assigned	88.3	2.94×10^{-3}
	$C_{17}H_{19}NO_3$	285.14	18.433	piperine[†]	not assigned	88.3	3.90×10^{-3}
	$C_{14}H_{32}N_2O_4$	292.24	0.943	tetrakis(2-hydroxypropyl)ethylenediamine	not assigned	88.2	2.03×10^{-3}
	$C_{17}H_{19}NO_3$	285.14	18.682	piperine[a]	not assigned	87.3	3.22×10^{-3}
	$C_9H_{11}NO_2$	165.08	1.525	L-phenylalanine	pharmaceuticals	89.3	7.42×10^{-3}
	$C_7H_8N_4O_2$	180.06	3.441	paraxanthine	stimulants	90.0	4.95×10^{-3}
	$C_8H_{10}N4O_2$	194.08	5.6	caffeine	stimulants	89.4	1.01×10^{-2}
	$C_6H_5NO_2$	123.03	0.983	nicotinic acid	tobacco	85.5	3.53×10^{-3}
S6	$C_{20}H_{30}O_2$	302.2	21.01	eicosapentaenoic acid[†]	fatty acid	92.8	1.80×10^{-3}
	$C_{20}H_{30}O_2$	302.2	21.14	eicosapentaenoic acid[†]	fatty acid	92.2	1.75×10^{-3}
	$C_{18}H_{30}O_2$	278.2	23.22	α-linolenic acid	fatty acid	92.0	1.96×10^{-3}
	$C_{20}H_{30}O_2$	302.2	25.63	eicosapentaenoic acid[†]	fatty acid	90.4	1.17×10^{-3}
	$C_{12}H_{18}O_3$	210.1	7.30	jasmonic acid	plant hormones	89.5	3.75×10^{-4}
	$C_{24}H_{30}O_6$	414.2	18.74	bis(4-ethylbenzylidene)sorbitol	industrial	92.2	1.76×10^{-3}
	$C_{16}H_{22}O_4$	278.2	21.19	dibutyl phthalate	industrial	89.6	5.45×10^{-3}
	$C_6H_{11}NO$	113.1	3.68	caprolactam	industrial	86.1	2.52×10^{-3}
	$C_{16}H_{13}N_3O_3$	295.1	13.54	mebendazole	pharmaceuticals	88.8	6.53×10^{-4}
	$C_{10}H_{13}N_5O_4$	267.1	1.07	adenosine	pharmaceuticals	97.3	5.88×10^{-3}
	$C_8H_9NO_2$	151.1	2.00	paracetamol[†]	pharmaceuticals	94.2	2.23×10^{-2}
	$C_8H_9NO_2$	151.1	1.88	paracetamol[†]	pharmaceuticals	94.1	8.08×10^{-3}
	$C_{21}H_{25}ClN_2O_3$	388.2	15.31	cetirizine	pharmaceuticals	92.4	7.07×10^{-4}
	$C_{32}H_{39}NO_4$	501.3	14.71	fexofenadine	pharmaceuticals	90.8	8.74×10^{-4}
	$C_{12}H_{15}N_3O_2S$	265.1	13.46	albendazole	pharmaceuticals	88.4	3.79×10^{-4}
	C_6H_9NOS	143.0	1.65	4-methyl-5-thiazoleethanol	pharmaceuticals	88.3	7.72×10^{-4}
	$C_{13}H_{12}F_2N_6O$	306.1	7.65	fluconazole	pharmaceuticals	88.3	6.78×10^{-4}
	$C_{10}H_{10}O_3$	178.1	25.38	4-methoxycinnamic acid	pharmaceuticals	85.8	1.48×10^{-3}
	$C_{17}H_{23}NO$	257.2	6.67	dextrorphan	pharmaceuticals	85.4	6.96×10^{-4}
	$C_9H_{11}NO_2$	165.1	1.51	L-phenylalanine[†]	pharmaceuticals	88.6	3.69×10^{-3}
	$C_9H_{11}NO_2$	165.1	1.42	L-phenylalanine[†]	pharmaceuticals	85.4	4.68×10^{-4}
	$C_{14}H_{22}N_2O_3$	266.2	1.92	atenolol	pharmaceuticals	85.0	2.55×10^{-3}

Table S11 (continued) - Compounds names, source categories and relative sample abundances of the compounds identified in the surface water samples using the commercial mass spectral library, mzCloud.

Sample ID	MF	MW	t_R	Name	Category	mzCloud match (%)	Normalized peak area*
S6	$C_8H_{10}N_4O_2$	194.1	5.61	caffeine	stimulants	93.6	1.78×10^{-2}
	$C_7H_8N_4O_2$	180.1	3.46	paraxanthine	stimulants	92.8	8.18×10^{-3}
	$C_6H_6N_4O_2$	166.0	1.64	1-methylxanthine†	stimulants	86.0	1.06×10^{-3}
	$C_6H_6N_4O_2$	166.0	1.78	1-methylxanthine†	stimulants	86.0	2.43×10^{-3}
	$C_{10}H_{12}N_2O$	176.1	1.14	cotinine	tobacco	91.0	7.97×10^{-3}
S7	$C_{24}H_{30}O_6$	414.20	18.73	bis(4-ethylbenzylidene)sorbitol	industrial	92.1	3.42×10^{-3}
	$C_{10}H_{13}N_5O_4$	267.10	1.058	adenosine	pharmaceuticals	97.4	2.61×10^{-2}
	$C_{17}H_{21}NO_3$	287.15	17.93	piperanine	pharmaceuticals	86.4	5.02×10^{-4}
	$C_4H_7N_3O$	113.06	0.754	creatinine	human/animal waste	97.4	2.32×10^{-2}
	$C_4H_7N_3O$	113.06	0.914	creatinine	human/animal waste	93.4	1.01×10^{-2}
	$C_{17}H_{19}NO_3$	285.14	18.305	piperine[a]	not assigned	88.6	2.56×10^{-3}
	$C_{17}H_{19}NO_3$	285.14	18.428	piperine[a]	not assigned	88.4	3.99×10^{-3}
	$C_{17}H_{19}NO_3$	285.14	18.677	piperine[a]	not assigned	86.9	3.99×10^{-3}
	$C_9H_{11}NO_2$	165.08	1.492	L-phenylalanine	pharmaceuticals	90.4	1.15×10^{-2}
	$C_{11}H_9NO_2$	187.06	2.525	indole-3-acrylic acid	plant hormones	87.1	2.67×10^{-3}
	$C_8H_{10}N_4O_2$	194.08	5.606	caffeine	stimulants	88.7	8.01×10^{-3}
	$C_7H_8N_4O_2$	180.06	3.463	paraxanthine	stimulants	87.9	2.98×10^{-3}
	$C_6H_5NO_2$	123.03	0.911	nicotinic acid	tobacco	89.6	4.44×10^{-3}

MF = molecular formula. MW = Molecular weight. t_R = retention time. * = Relative sample peak area. † = Suspected structural isomers of duplicate compound.

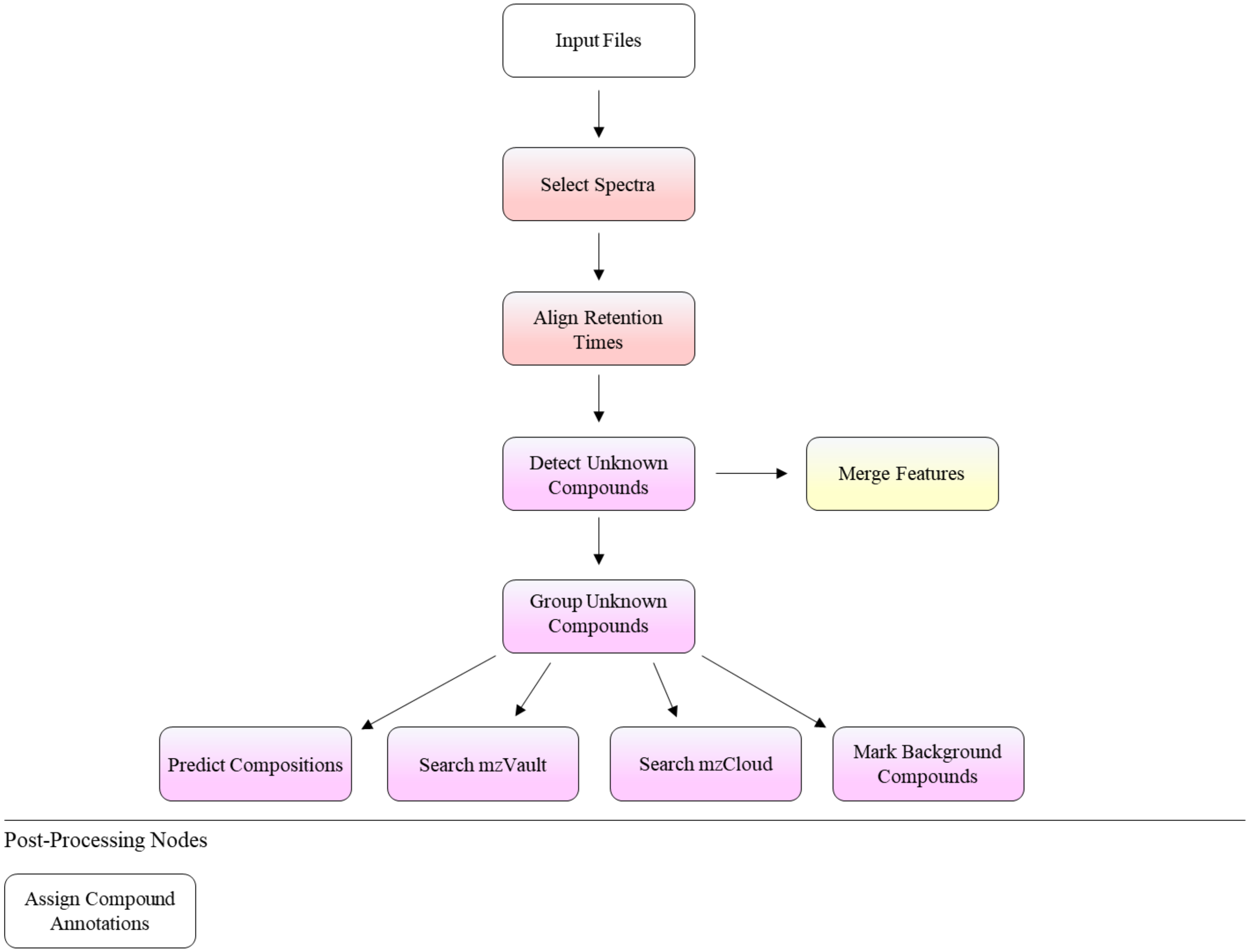

Figure S1 – A schematic of the bespoke non-targeted workflow developed in Compound Discoverer. Coloring corresponds to the node groupings presented in the software.

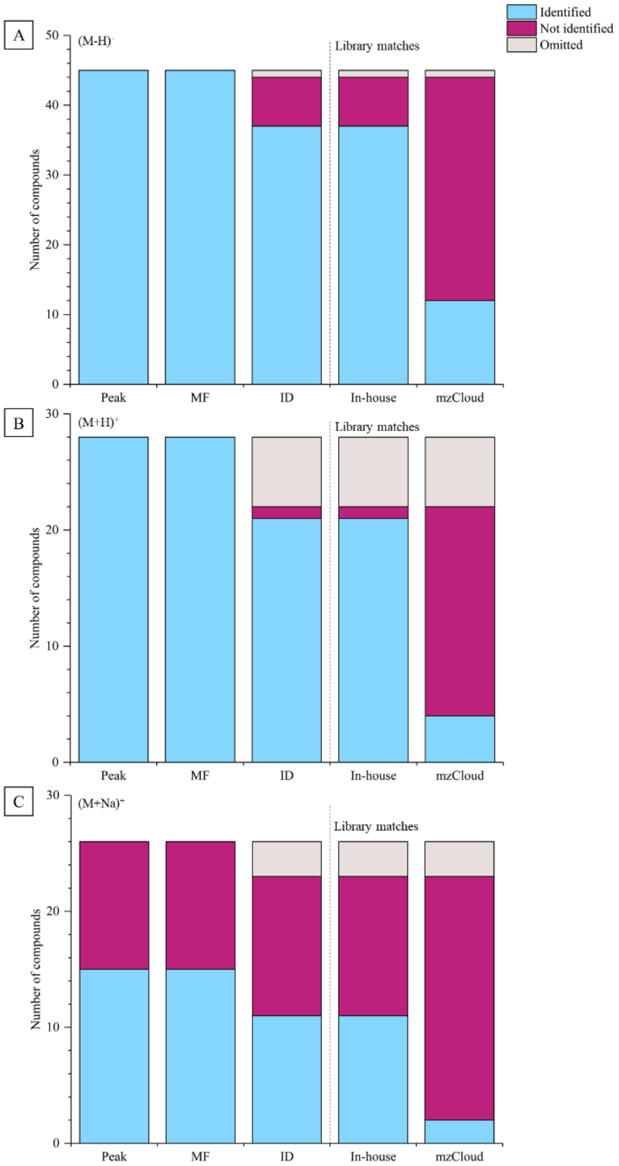

Figure S2 – Performance of the non-targeted method to detect and identify 60 individually prepared standards at a concentration of 1 ppm (see manuscript section ‘Initial Software Testing’ for further information). Each plot displays whether the chromatographic peak (Peak), molecular formulae (MF) and compound name (ID) were correctly identified by the non-targeted method for $[M\text{-}H]^-$ (A), $[M+H]^+$ (B) and $[M+Na]^+$ (C). A correct identification was reported if the non-targeted method reported the same result as manual data processing. Compound names were assigned using the in-house or commercial (mzCloud) MS^2 library. The number of compounds identified using each MS^2 library is shown (‘library matches’). Omitted data = no MS^2 data recorded during analysis, preventing molecular identification.

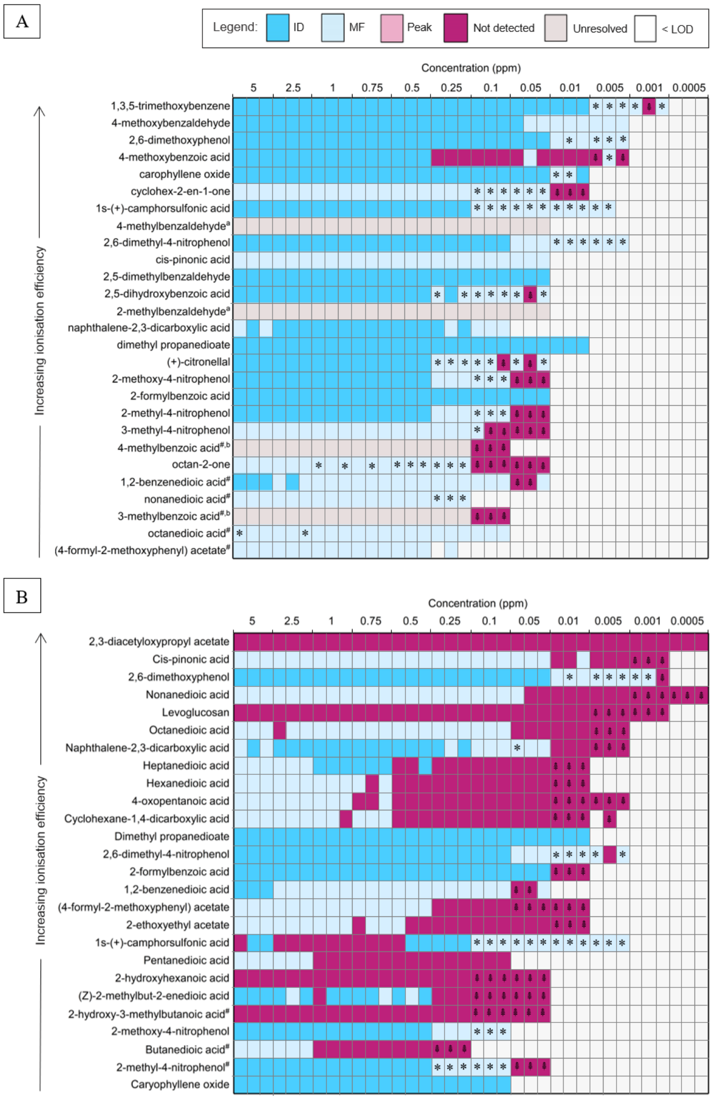

Figure S3 – Performance of the non-targeted method to detect and identify (A) $[M+H]^+$ and (B) $[M+Na]^+$ in the standard mix at various concentrations. Plot displays whether the compound names (ID), molecular formulae (MF) and chromatographic peak (Peak) were identified. Each box represents one measurement, with 3 replicate sample injection measurements and data analyses performed for each concentration. * = No MS^2 data acquired during analysis preventing molecular identification. ⸙ = Chromatographic peak cannot be detected due to the use of unit or near unit mass resolution. Isomeric species which could not be resolved *via* manual or automated data processing are shown in grey. Letters correspond to the groups of isomeric species which could not be resolved; ^{a} = 2-methylbenzaldhyde and 4-methylbenzaldhyde. ^{b} = 3-methylbenzoic acid and 4-methylbenzoic acid. $^\#$ = In-house library contains no MS^2 spectra for this standard.

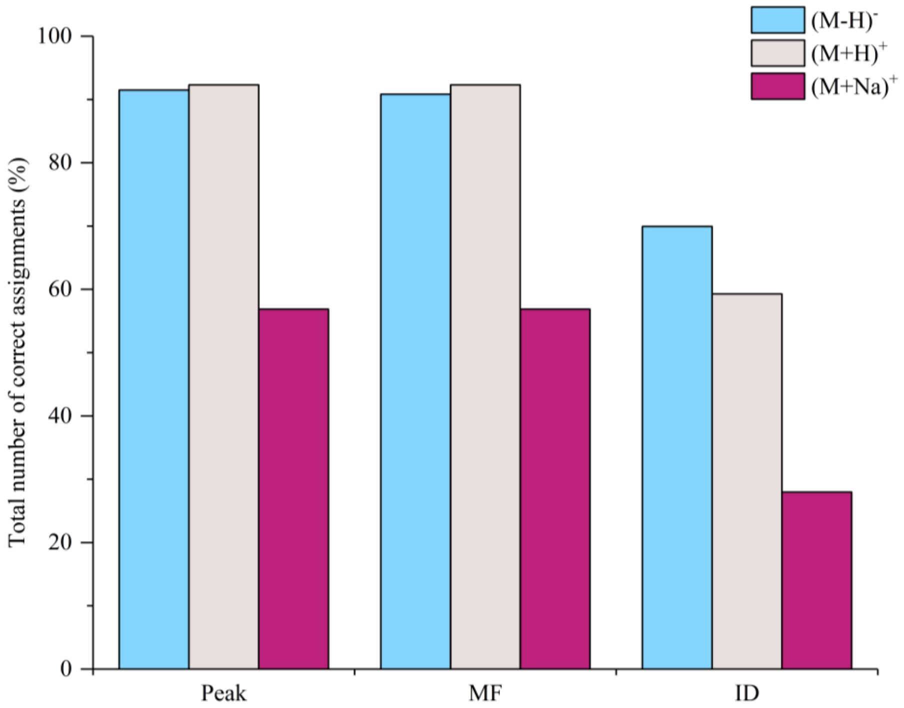

Figure S4 – Performance of the non-targeted screening method to correctly identify the chromatographic peak (‘Peak’), molecular formula (‘MF’) and chemical identity (‘ID’) of each molecular species in the standard mixtures at concentrations ranging from 5 ppm to 0.05 ppb. Plot provides the total number of correct assignments (in percentage) observed in Figures 1 and S3.

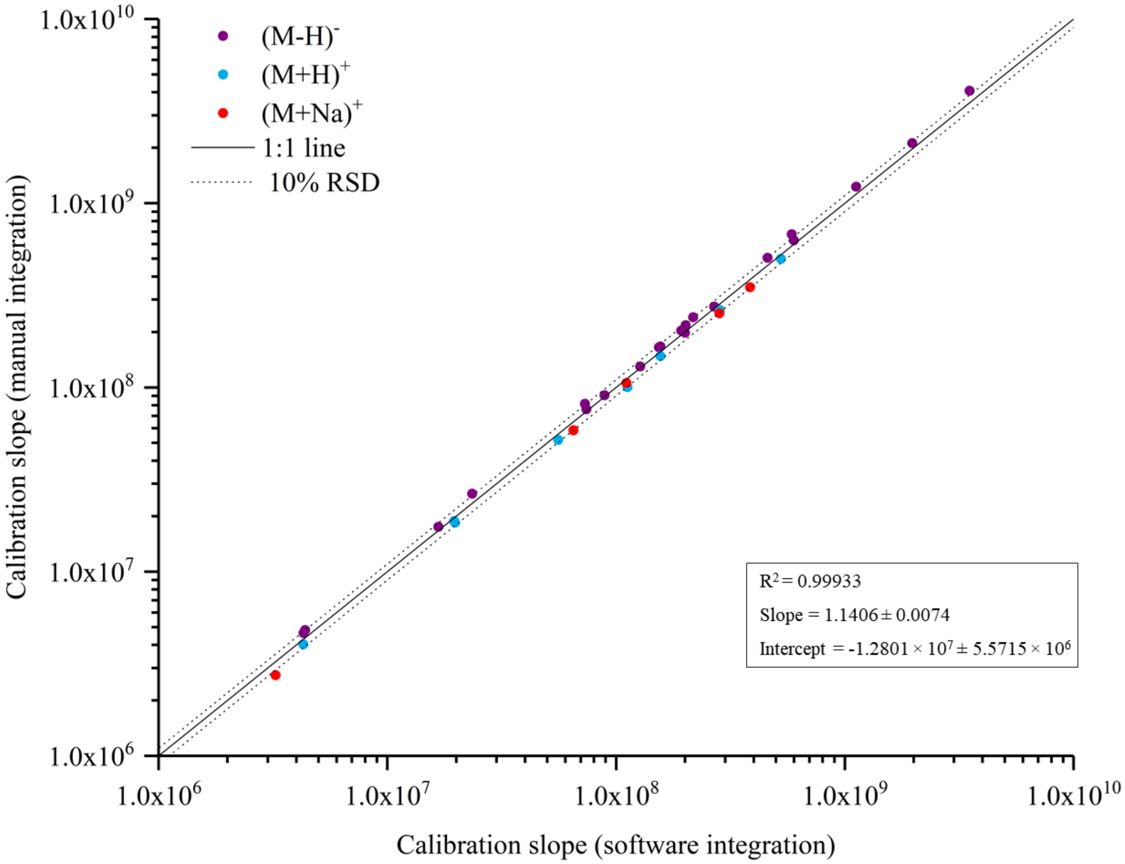

Figure S5 – Performance of non-targeted method *vs.* manual analysis for chromatographic peak integration. Plot shows the calibration slope of a detected molecular species integrated manually ('manual integration'), divided by the calibration slope of the same molecular species integrated using the non-targeted method ('software integration'). Each calibration graph consisted of a minimum of 5 concentrations and 3 replicate measurements per concentration.

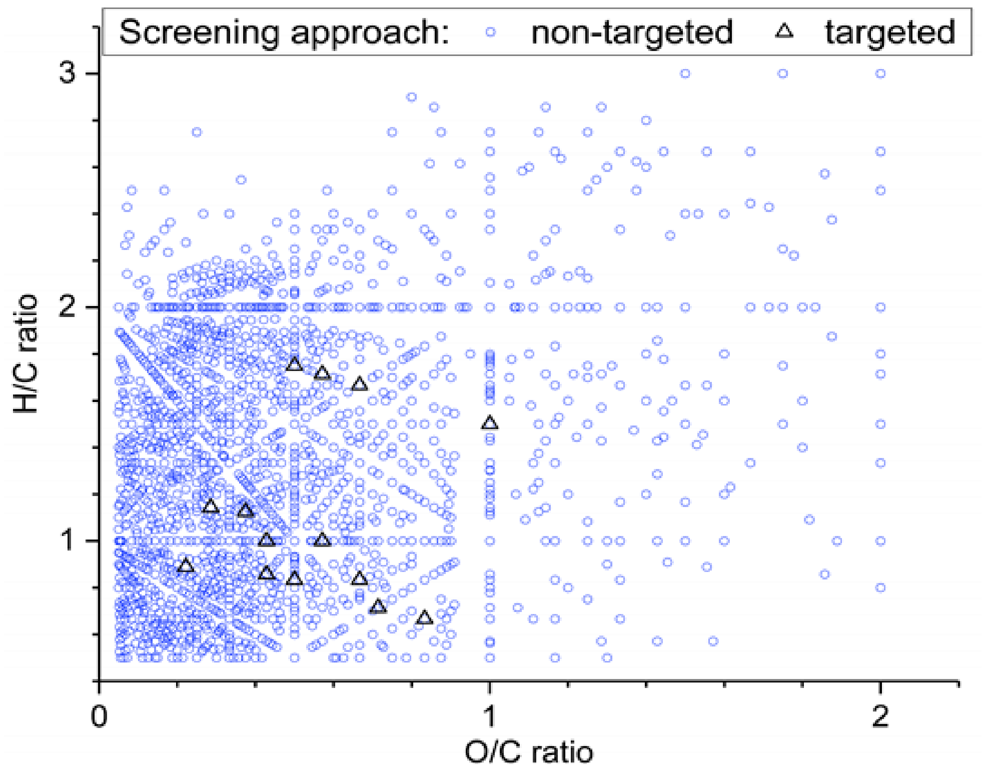

Figure S6 - A comparison of the number of compounds detected in one PM sample (sample 96, see Table S2) using the targeted and non-targeted screening approach in negative ionisation mode, shown in a chemical space. Each symbol corresponds to one molecular formula, representing in some cases, multiple compounds (isomers). 60 environmental compounds were targeted, only 20 were detected (see Table S1 for the targeted compounds). In contrast, the non-targeted screening approach detected 5089 unique compounds (*i.e.* chemically and/or structurally different).

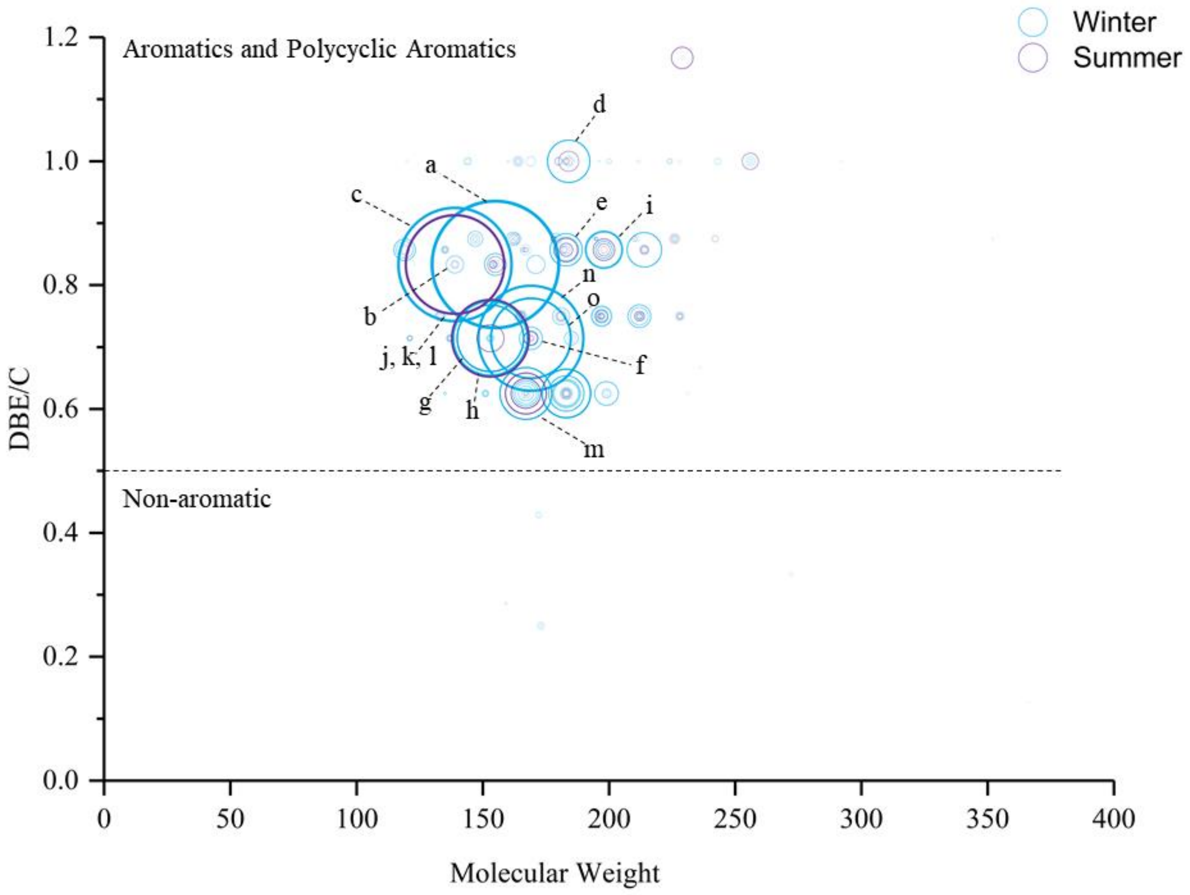

Figure S7 – Comparison of CHON C_6 to C_8 containing species in the winter (sample = 94, see Table S2) and summer (sample = 261) $PM_{2.5}$ samples. Each bubble represents one compound, and the size of the bubble displays the normalized sample peak area of the compound in each sample. Letters correspond to molecular identifications using the tandem mass spectral libraries; a = 4-nitrobenzene-1,2-diol, b = 3-nitrophenol, c = 4-nitrophenol, d = 2,4-dinitrophenol, e = 2-hydroxy-5-nitrobenzoic acid, f = 4-nitroguaiacol, g = 3-methyl-4-nitrophenol, h = 2-methyl-4-nitrophenol, i = 3,5-dintro-o-cresol, j = 5-hydroxyindole, k= isomer of 5-hydroxyindole, l = isomer of 5-hydroxyindole and, m = 2,6-dimethyl-4-nitrophenol. Letters n and o display the suspected methyl nitrocatechols at t_R 8.82 and 7.15, respectively.

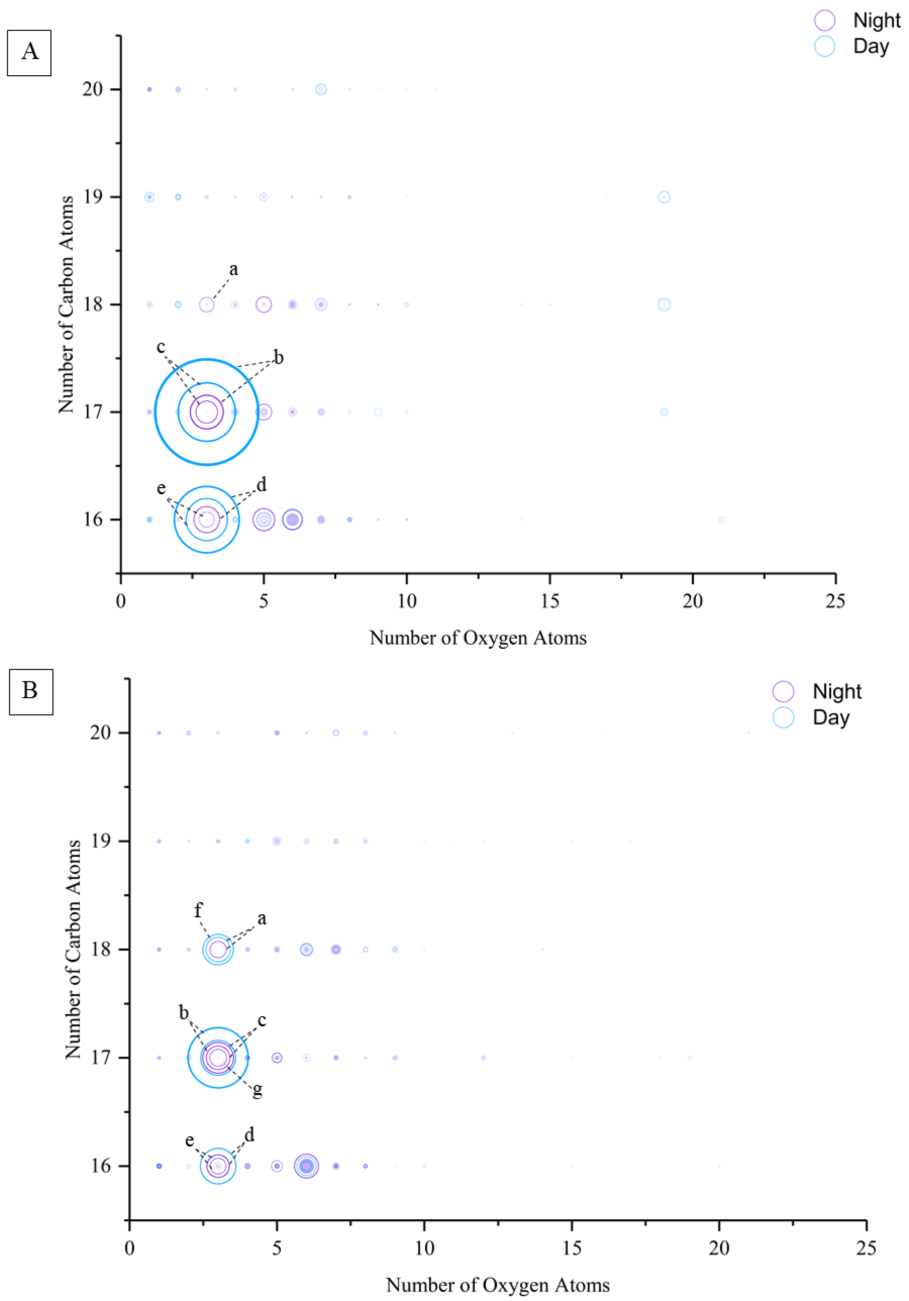

Figure S8 – Comparison of the C_{16} to C_{20} CHOS species in the $PM_{2.5}$ samples collected in the summer (A) and winter (B) seasons. Each circle represents one compound and the size of the circle represent the normalized sample peak area. Figure A displays samples 261 (daytime) and 264 (nighttime), see SI Table S2. Figure B displays samples 94 (daytime) and 96 (nighttime). a = 4-dodecylbenzenesulfonic acid, b = $C_{17}H_{28}O3_{S}$ t_R 22.37, c = $C_{17}H_{28}O3_{S}$ t_R 22.68, d = $C_{16}H_{26}O_3S$ t_R 21.25, e = $C_{16}H_{26}O_3S$ t_R 21.53, f = $C_{18}H_{30}O_3S$, t_R 23.73 (suspected 4-dodecylbenzenesulfonic acid structural isomer) and g = $C_{17}H_{28}O3_{S}$ t_R 22.49.

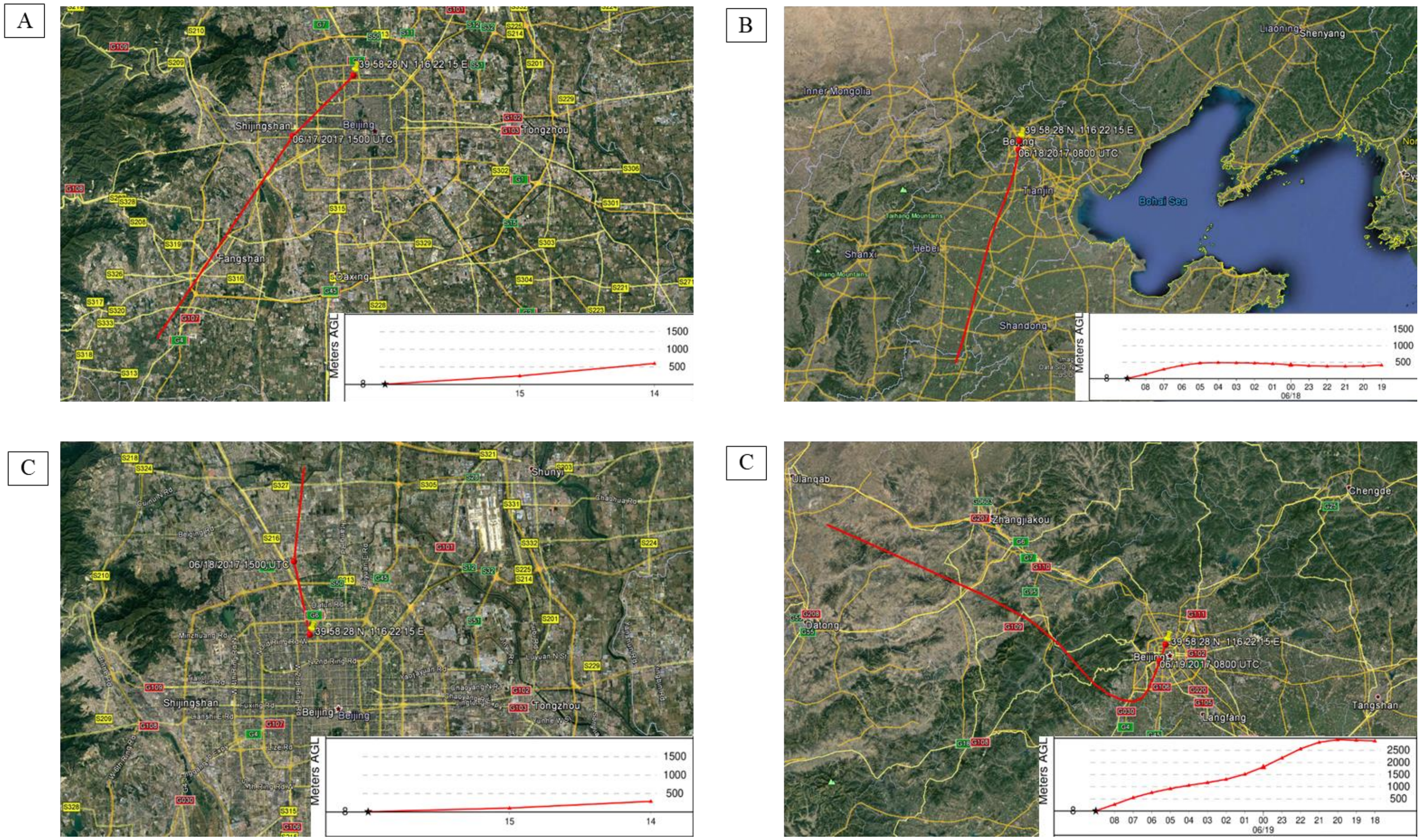

Figure S9 – Back-trajectory modelled data showing the transport of the sampled air masses during the summer season for (A) sample 261 (17/06/17, day), (B) sample 264 (17/06/17, night), (C) sample 271 (18/06/17, day) and (D) sample 274 (18/06/17, night). The sampling site is shown by the yellow pin. The red line on the map shows the air mass transport. The Figure in the bottom right corner shows the height of the air mass transport (*y*-axis, meters) *vs.* the sampling date and time (hours). An 8 meter height was set at the sampling location corresponding to the sampler height (see Materials and Methods). Data was calculated using the HYSPLIT trajectory model provided by NOAA (https://ready.arl.noaa.gov/HYSPLIT_traj.php).

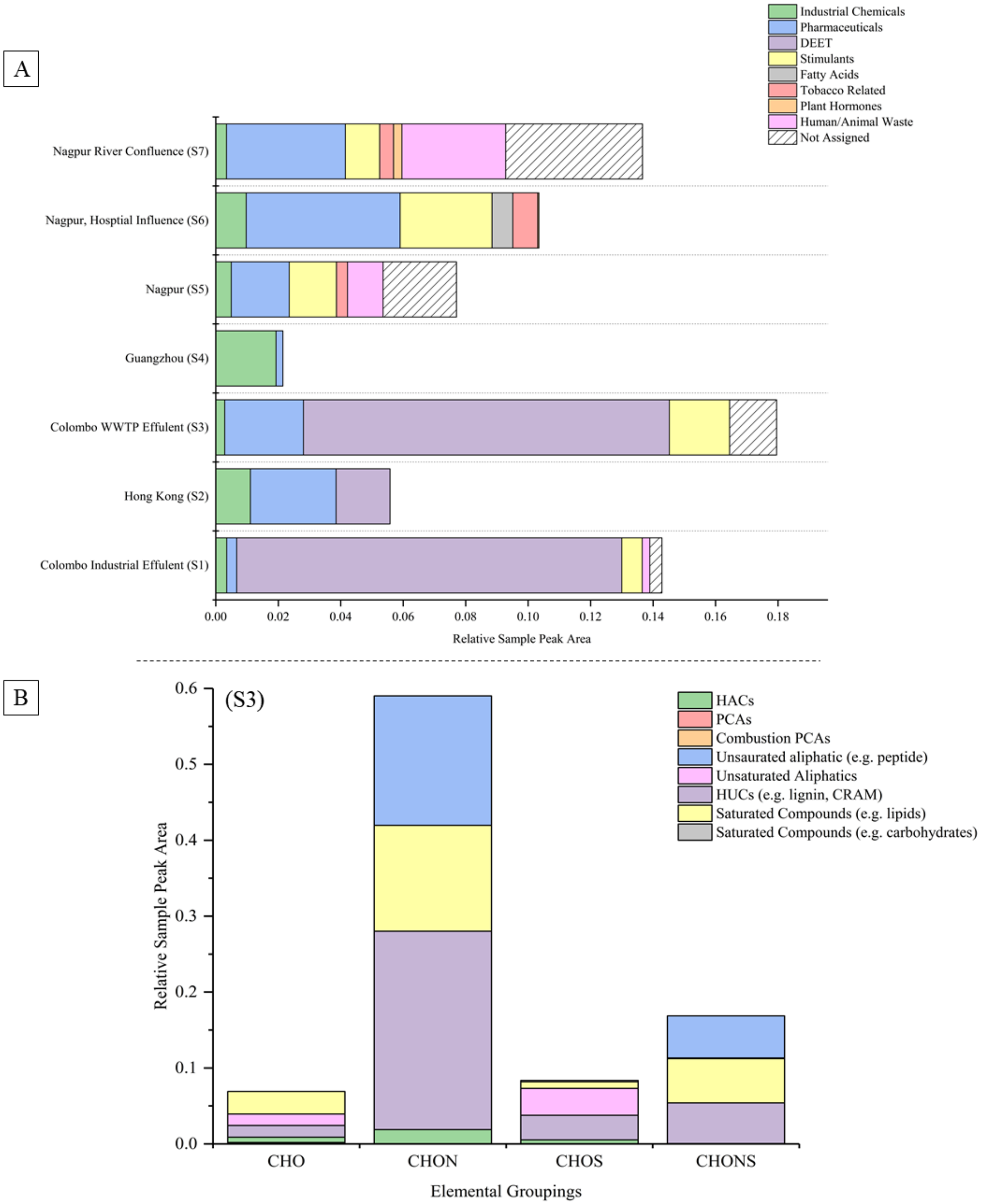

Figure S10 – The categorized pollutant sources of the compounds tentatively identified using mzCloud with spectral matches >85% confidence (A) and the DOM chemical groupings of wastewater treatment plant (WWTP) effluent collected in Colombo, Sri Lanka (sample S3, see Table S3) (B). Further information regarding the pollutant groupings can be found in the manuscript text. The total number of identified compounds and their assigned pollutant groupings can be found in Table S11.

References

1. Gao, S.; Surratt, J. D.; Knipping, E. M.; Edgerton, E. S.; Shahgholi, M.; Seinfeld, J. H., Characterization of polar organic components in fine aerosols in the southeastern United States: Identity, origin, and evolution. *Journal of Geophysical Research: Atmospheres* **2006,** *111*, (D14).

2. Hamilton, J. F.; Lewis, A. C.; Carey, T. J.; Wenger, J. C., Characterization of Polar Compounds and Oligomers in Secondary Organic Aerosol Using Liquid Chromatography Coupled to Mass Spectrometry. *Analytical Chemistry* **2008,** *80*, (2), 474-480.

3. Kourtchev, I.; Godoi, R. H. M.; Connors, S.; Levine, J. G.; Archibald, A. T.; Godoi, A. F. L.; Paralovo, S. L.; Barbosa, C. G. G.; Souza, R. A. F.; Manzi, A. O.; Seco, R.; Sjostedt, S.; Park, J. H.; Guenther, A.; Kim, S.; Smith, J.; Martin, S. T.; Kalberer, M., Molecular composition of organic aerosols in central Amazonia: an ultra-high-resolution mass spectrometry study. *Atmos. Chem. Phys.* **2016,** *16*, (18), 11899-11913.

4. Donahue, N.; Chuang, W.; Ortega, I. K.; Riipinen, I.; Riccobono, F.; Schobesberger, S.; Dommen, J.; Kulmala, M.; Worsnop, D.; Vehkamaki, H., How Do Organic Vapors Contribute to New-Particle Formation? *Faraday Discuss.* **2013**.

5. Kalberer, M.; Paulsen, D.; Sax, M.; Steinbacher, M.; Dommen, J.; Prevot, A.; Fisseha, R.; Weingartner, E.; Frankevich, V.; Zenobi, R., Identification of polymers as major components of atmospheric organic aerosols. *Science* **2004,** *303*, (5664), 1659-1662.

6. Koch, B. P.; Dittmar, T., From mass to structure: an aromaticity index for high-resolution mass data of natural organic matter. *Rapid Communications in Mass Spectrometry* **2006,** *20*, (5), 926-932.

7. Melendez-Perez, J. J.; Martínez-Mejia, M. J.; Eberlin, M. N., A reformulated aromaticity index equation under consideration for non-aromatic and non-condensed aromatic cyclic carbonyl compounds. *Organic Geochemistry* **2016,** *95*, 29-33.

8. Kroll, J. H.; Donahue, N. M.; Jimenez, J. L.; Kessler, S. H.; Canagaratna, M. R.; Wilson, K. R.; Altieri, K. E.; Mazzoleni, L. R.; Wozniak, A. S.; Bluhm, H.; Mysak, E. R.; Smith, J. D.; Kolb, C. E.; Worsnop, D. R., Carbon oxidation state as a metric for describing the chemistry of atmospheric organic aerosol. *Nat Chem* **2011,** *3*, (2), 133-139.

9. Stenson, A. C.; Marshall, A. G.; Cooper, W. T., Exact Masses and Chemical Formulas of Individual Suwannee River Fulvic Acids from Ultrahigh Resolution Electrospray Ionization Fourier Transform Ion Cyclotron Resonance Mass Spectra. *Analytical Chemistry* **2003,** *75*, (6), 1275-1284.

10. Hertkorn, N.; Benner, R.; Frommberger, M.; Schmitt-Kopplin, P.; Witt, M.; Kaiser, K.; Kettrup, A.; Hedges, J. I., Characterization of a major refractory component of marine dissolved organic matter. *Geochimica et Cosmochimica Acta* **2006,** *70*, (12), 2990-3010.
